# Characterization of gamma irradiation-induced mutations in Arabidopsis mutants deficient in non-homologous end joining

**DOI:** 10.1093/jrr/rraa059

**Published:** 2020-08-07

**Authors:** Yan Du, Yoshihiro Hase, Katsuya Satoh, Naoya Shikazono

**Affiliations:** 1 Biophysics Group, Institute of Modern Physics, Chinese Academy of Sciences, Lanzhou 730000, PR China; 2 Takasaki Advanced Radiation Research Institute, National Institutes for Quantum and Radiological Science and Technology (QST), 1233 Watanuki, Takasaki, Gunma 370-1292, Japan; 3 Kansai Photon Science Institute, National Institutes for Quantum and Radiological Science and Technology (QST), 1233 Watanuki, Takasaki, Gunma 370-1292, Japan

**Keywords:** Arabidopsis, *Ku70*, *Lig4*, NHEJ, gamma ray, mutation

## Abstract

To investigate the involvement of the non-homologous end joining (NHEJ) pathway in plant mutagenesis by ionizing radiation, we conducted a genome-wide characterization of the mutations induced by gamma rays in NHEJ-deficient Arabidopsis mutants (*AtKu70*^−/−^ and *AtLig4*^−/−^). Although both mutants were more sensitive to gamma rays than the wild-type control, the *AtKu70*^−/−^ mutant was slightly more sensitive than the *AtLig4*^−/−^ mutant. Single-base substitutions (SBSs) were the predominant mutations in the wild-type control, whereas deletions (≥2 bp) and complex-type mutations [i.e. more than two SBSs or short insertion and deletions (InDels) separated by fewer than 10 bp] were frequently induced in the mutants. Single-base deletions were the most frequent deletions in the wild-type control, whereas the most common deletions in the mutants were 11–30 bp. The apparent microhomology at the rejoined sites of deletions peaked at 2 bp in the wild-type control, but was 3–4 bp in the mutants. This suggests the involvement of alternative end joining and single-strand annealing pathways involving increased microhomology for rejoining DNA ends. Complex-type mutations comprising short InDels were frequently detected in the mutants, but not in the wild-type control. Accordingly, NHEJ is more precise than the backup pathways, and is the main pathway for rejoining the broken DNA ends induced by ionizing radiation in plants.

## INTRODUCTION

A thorough characterization of the DNA repair mechanism in plants is important for elucidating the maintenance of genome integrity and for improving crops via mutagenesis or genetic modification. Homologous recombination (HR) and non-homologous end joining [NHEJ, also known as canonical- or classical-NHEJ (c-NHEJ)] are two major and mechanistically distinct pathways in double-strand break (DSB) repair [1–3]. Additionally, HR is a high-fidelity repair pathway mediated by several components, including RAD51, RAD54, BRCA1 and BRCA2, and occurs only in the S and G_2_ phases because it requires a homologous template (e.g. a sister chromatid) [4, 5]. In contrast, NHEJ relies on considerably less or no homology, and directly ligates DNA ends with high fidelity or small alterations at the junctions [6–8]. In NHEJ, the DSBs are first recognized by a Ku70/Ku80 heterodimer, after which the DNA ends are processed by the related nucleases and polymerases to generate a ligatable configuration. The DNA ligase 4 (Lig4) complex ligates the ends of either strand to complete the repair of the DSB. Two other pathways, namely alternative end joining (alt-EJ) and single-strand annealing (SSA), also contribute to DSB repair. Specifically, alt-EJ is a Ku protein-independent end joining pathway, with poly (ADP-ribose) polymerase 1 (PARP1) and DNA polymerase theta as core components. The RAD52-mediated annealing of homologous sequences is a key process for SSA. Alt-EJ and SSA generally require longer microhomology than NHEJ for rejoining [1]. Moreover, alt-EJ and SSA are non-conservative repair pathways because they need extensive end resectioning to generate ligatable single-strand DNA ends.

In higher eukaryotes, NHEJ is thought to be the predominant pathway of DSB repair [9]. In plants, the characteristics of induced mutations in NHEJ-deficient mutants have been analyzed to enhance the efficiency of genome editing because genome-editing technologies rely on the misrepair of DNA cleavage by site-directed artificial nucleases. Osakabe *et al*. [10] revealed that deletions induced by zinc finger nucleases are 3 bp or shorter in wild-type Arabidopsis, whereas the number of deletions longer than 4 bp substantially increases in a *Ku80*-deficient background. In a similar study involving *ku70* and *lig4* Arabidopsis mutants, deletions were mainly shorter than 10 bp in the wild-type control, but were usually 10–49 bp in the mutants [11]. Additionally, nearly 90% of the deletions had no or 1 bp of microhomology at the rejoined sites in the wild-type control, whereas 1–6 bp microhomologies were common in the *ku70* and *lig4* mutants. Consistent results were obtained using a clustered regularly interspaced short palindromic repeats (CRISPR)/CRISPR-associated protein 9 (Cas9) system and *ku70* and *ku80* Arabidopsis mutants [9, 12]. The enhanced frequency of targeted mutagenesis and the extensive use of microhomology for DSB repair were also reported for *Lig4*-deficient rice [13]. These studies suggest that in the absence of NHEJ, DSBs are repaired by alt-EJ, SSA, or other backup pathways that rely on more extensive homology and tend to cause larger deletions.

Ionizing radiation, including gamma rays, have been widely used as powerful mutagens in plants. Compared with enzymatically-induced DSBs, DNA damage caused by ionizing radiation are more complex regarding the types of damage (e.g., single- or double-strand breaks, base damage and abasic sites) as well as their combination and spatial distribution [14]. Additionally, the complexity and proximity of DNA damage increase as the linear energy transfer (LET) of the radiation increases [15]. Whole-genome sequencing technology has recently been applied to characterize the mutations induced by various types of ionizing radiation [16–24]. Generally, regardless of the plant species and radiation type, single-base substitutions (SBSs) are the most frequent mutations. Moreover, the frequency and size of insertion and deletion (InDel) mutations and the occurrence of chromosome structural alterations increase with increasing LET. To date, ionizing radiation-induced mutations in an NHEJ-deficient background have not been fully characterized in plants. Consequently, how NHEJ influences the mutations generated by ionizing radiations remains unclear. In the present study, we examined the genome-wide mutations caused by gamma rays in Arabidopsis mutants deficient in *Ku70* and *Lig4*. The resulting data were compared with those of the wild-type control.

## MATERIALS AND METHODS

### Plant materials and growth conditions

*Arabidopsis thaliana* seeds with T-DNA insertions in *AtKu70* (At1g16970, SALK_123114) and *AtLig4* (At5g57160, SALK_044027) were obtained from the Arabidopsis Biological Resource Center (Ohio State University, Columbus, OH). Plant genotypes were identified by polymerase chain reaction (PCR) using a T-DNA border-specific primer and gene-specific genomic primers (Supplementary Figure S1, see online supplementary material). Seeds collected from a single homozygous plant were used to minimize the background mutations among the individual plants of each line. Seeds were sown in a pot filled with a 1:1 mixture of culture soil (TM-2, Takii & Co., Ltd. Japan) and vermiculite (medium size, Vern-piece; Hakugen Co., Ltd., Tokyo, Japan) and then incubated in a growth room (Koito Industries, Yokohama, Japan) at 23 °C with a 16-h light/8-h dark photoperiod, unless otherwise indicated.

### Gamma irradiation

Dry seeds were exposed to ^60^Co gamma rays (50–350 Gy) at the Takasaki Advanced Radiation Research Institute, National Institutes for Quantum and Radiological Science and Technology. The survival rate of the irradiated seeds was determined at 1 month after sowing. Seedlings with more than five fresh and viable rosette leaves (true leaves) were counted as survivors. Survival curves were drawn based on the single hit-multitarget theory as previously described [24]. The M_2_ seeds were harvested from individual plants.

Regarding seedling irradiation, dry seeds were surface-sterilized with bleach for 10 min, washed with sterile water five times, and then sown on half-strength Murashige and Skoog (MS) medium in plates. After a 3-day vernalization at 4 °C in darkness, the plates were transferred to the growth room and positioned vertically. One week later, the seedlings were exposed to gamma rays (50 Gy for the wild-type control and 10 Gy for the mutant lines). Seedlings were harvested at 0 (non-irradiated control), 6, 12 and 24 h after the irradiation, immediately frozen in liquid nitrogen and stored at −80 °C.

### Whole-genome re-sequencing

Genomic DNA was isolated from a single randomly selected plant for each M_2_ line with a MagExtractor Plant Genome DNA Extraction Kit (Toyobo Co. Ltd., Tokyo, Japan). Sequencing libraries were prepared with a Nextera DNA Flex Library Prep kit and the IDT for Illumina Nextera DNA UD Indexes Set A (Illumina, Inc. USA) according to the manufacturer’s instructions. The libraries were sequenced with an Illumina NextSeq 500 platform to generate 150-bp paired-end reads.

### Mutation detection

The raw sequence reads were processed and mapped to the Arabidopsis reference genome (TAIR10), and candidate mutation sites were identified using GATK Haplotype Caller (version 3.4, https://software.broadinstitute.org/gatk/), Pindel (version 0.2.4, http://gmt.genome.wustl.edu/packages/pindel/user-manual.html) and BreakDancer (version 1.4.5, http://breakdancer.sourceforge.net/) algorithms as previously described [24]. All mutations assayed are in unique regions, since we used the golden path of chromosomes 1–5 (119, 146, 348 bp) as a reference sequence, which does not contain redundant regions. The average depth of coverage was 24–75× and ≥ 97% of the target bases were covered at a minimum of 10× (Supplementary Table S1, see online supplementary material). The candidate mutation sites detected by GATK in more than two independent samples were excluded as false positives. The candidate mutation sites with allele frequencies (AF; proportions of mutant reads at a site) ≤ 25% were also excluded. The mutation site was considered heterozygous if 25% < AF < 80% and AF < 5% for all other samples. The candidate mutation site was considered homozygous if AF ≥ 80% and AF < 5% for all other samples. All candidate mutations were confirmed with Integrative Genomics Viewer (IGV; version 2.7.1; http://software.broadinstitute.org/software/igv/). For the Pindel and BreakDancer analyses, the candidate mutation sites unique to a single sample were selected and confirmed with IGV. The sequence of each read was also confirmed as necessary.

### Sanger sequencing

A total of 34 mutations that covered all mutation types were randomly selected. Primers were designed with Primer3 (version 0.4.0; http://bioinfo.ut.ee/primer3-0.4.0/), after which their specificity was evaluated with MFEprimer-3.0 (https://www.mfeprimer.com/). Sequence details for all primers are listed in Supplementary Table S2, see online supplementary material. The same DNA samples used for whole-genome re-sequencing were used as PCR templates. The amplified fragments with the expected length were purified with a MinElute PCR Purification Kit (Qiagen K. K., Tokyo, Japan) and sequenced with a BigDye Terminator (version 3.1) Cycle Sequencing Kit (Thermo Fisher Scientific K. K., Yokohama, Japan) and a 3500 Genetic Analyzer (Applied Biosystems, Tokyo, Japan) according to the manufacturers’ instructions.

### Quantitative reverse-transcription PCR

Total RNA was isolated from irradiated seedlings with an RNeasy Plant Mini Kit (Qiagen) and then used as the template to synthesize cDNA with a PrimeScript RT-PCR kit (Takara, Shiga, Japan) according to the manufacturers’ instructions. A quantitative PCR assay was completed with a CFX 96 Real-Time PCR system (Bio-Rad, Foster City, CA) and a KAPA SYBR Fast qPCR kit (KAPA Biosystems, Wilmington, MA). The PCR program was as follows: 95 °C for 3 min; 40 cycles of 95 °C for 3 s and 60 °C for 20 s. The PCR primers (Supplementary Table S3, see online supplementary material) were designed with the OligoArchitect online program (http://www.oligoarchitect.com/LoginServlet). The *ACTIN2* gene (At3g18780) was used as an endogenous control for normalizing expression data.

### Statistical analysis and data availability

Data were analyzed by a one-way ANOVA with a multiple comparison test or *t*-test based on the number of biological replicates. A *P*-value < 0.05 was applied as the significance threshold. The whole-genome re-sequencing data were deposited in the DNA Data Bank of Japan Sequence Read Archive (http://ddbj.nig.ac.jp/dra) with accession number DRA010046.

## RESULTS

### Sensitivity to gamma irradiation

**Fig. 1.**
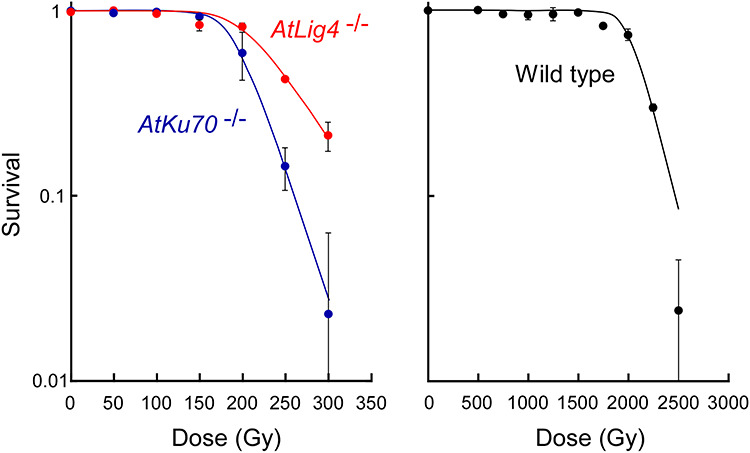
Survival rate of dry seeds irradiated with gamma rays. Data are presented as the mean ± standard error of three replications of 30 plants. Data for the wild-type control are from a previous study by Hase *et al*. [24]. The survival curves were drawn based on a single hit-multitarget theory.

To examine the sensitivity of NHEJ-deficient Arabidopsis mutants to radiation, the dry seeds of homozygous *AtKu70* (*AtKu70*^−/−^) and *AtLig4* (*AtLig4*^−/−^) mutant lines were treated with gamma rays. The mutant lines were considerably more sensitive to the radiation than the wild-type control (Fig. 1). The doses corresponding to the shoulder of the survival curve (*Dq*) were 222 Gy for *AtLig4*^−/−^ and 193 Gy for *AtKu70*^−/−^, whereas it was 2045 Gy for the wild-type control. The *AtKu70*^−/−^ mutant appeared to be more sensitive than the *AtLig4*^−/−^ mutant, with a significantly lower survival rate at 250 and 300 Gy (*t*-test, *P* < 0.05). Both mutant lines were treated with 100 Gy and then compared with the previously reported wild-type plants gamma-irradiated with 1000 Gy [24]. These doses correspond to ~50% *Dq* for each material. Obvious abnormal morphologies were observed in the M_1_ generation at the selected doses (e.g. small plants and compact leaf arrangement), although the survival rate exceeded 94% at these doses. The M_2_ seeds were harvested from individual M_1_ plants.

### Frequency and types of mutations

Ten *AtKu70*^−/−^ and nine *AtLig4*^−/−^ M_2_ plants were analyzed by whole-genome re-sequencing. The detected mutations were classified into the following seven categories as previously described [24]: SBS, single-base deletion (−1), single-base insertion (+1), deletion of two or more base pairs (Del ≥2 bp), insertion of two or more base pairs (Ins ≥2 bp), complex-type and structural variant (SV). The complex-type mutations (i.e. more than two mutations separated by fewer than 10 bp) were considered to have resulted from a single event. Two or more bases identical to the reference genome were assumed to represent a non-mutated sequence. The SV category comprised inversions and translocations. We verified a part of the detected mutations by PCR and Sanger sequencing in order to validate our mutation detection method (Supplementary Table S2 and Supplementary Figure S2, see online supplementary material). The ratio of homozygous to heterozygous mutations did not differ significantly among the strains or from the theoretically expected ratio of 0.5 in the M_2_ generation (Supplementary Table S4, see online supplementary material). Therefore, the detected mutations were characterized irrespective of the zygosity. Since we detected mutations in M_2_ generation, non-transmissible mutations such as extremely large deletions and translocations, which may have been generated in M_1_ generation, are not included.

**Fig. 2.**
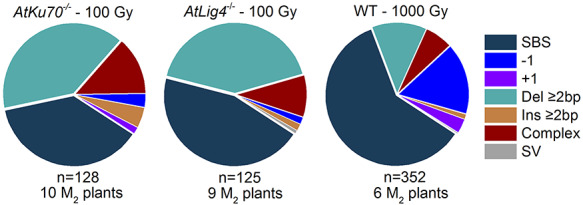
Mutation types induced by gamma rays. The numbers below the pie charts indicate the total number of mutation events and the number of examined M_2_ plants. Data for the wild-type control are from a previous study by Hase *et al*. [24].

With the radiation doses applied in this study, fewer total mutations were detected in the *AtKu70*^−/−^ and *AtLig4*^−/−^ mutants than in the wild-type control (Table 1). The total number of mutation events actually observed per plant was 49.10 in the wild-type control and 10.71−11.62 in the NHEJ-deficient mutants. However, on a per unit dose basis, there were more than twice as many mutations in the NHEJ-deficient mutants than in the wild-type control (Table 1). There were marked differences between the wild-type control and mutants in terms of the mutation types. Specifically, SBSs were the most frequent mutations in the wild-type control (62% of the total mutations), followed by the −1 (14%) and Del ≥2 bp (13%) mutations (Fig. 2). In the NHEJ-deficient mutants, SBS (38–45%) and Del ≥2 bp (40–42%) were the predominant mutations, followed by the complex-type mutations (10−13%). These results suggest that when NHEJ is compromised, more than twice as many mutations with a higher fraction of Del ≥2 bp and complex-type mutations occur per unit dose.

**Table 1:** Frequency of each mutation type^a^; data are presented as the mean ± standard error

Mutation/bp (×10^−8^)								
	SBS	–1	+1	Del ≥2 bp	Ins ≥2 bp	Complex	SV	Total
*AtKu70^−/−^* − 100 Gy	4.02 ± 0.46	0.34 ± 0.14	0.17 ± 0.11	4.27 ± 0.55	0.5 ± 0.19	1.42 ± 0.25	ND	10.71 ± 0.61
*AtLig4^−/−^* − 100 Gy	5.21 ± 0.95	0.19 ± 0.12	ND	4.84 ± 0.91	0.19 ± 0.12	1.12 ± 0.28	0.09 ± 0.09	11.62 ± 1.97
WT^b^—1000 Gy	30.27 ± 3.36	6.98 ± 1.20	1.67 ± 0.89	6.28 ± 0.74	0.56 ± 0.28	3.21 ± 0.40	0.14 ± 0.14	49.10 ± 4.36
Mutation/bp/Gy (×10^−10^)								
	SBS	–1	+1	Del ≥2 bp	Ins ≥2 bp	Complex	SV	Total
*AtKu70^−/−^*	4.02 ± 0.46	0.34 ± 0.14	0.17 ± 0.11	4.27 ± 0.55^*^^*^	0.5 ± 0.19	1.42 ± 0.25^*^^*^	ND	10.71 ± 0.61^*^^*^
*AtLig4^−/−^*	5.21 ± 0.95	0.19 ± 0.12^*^	ND	4.84 ± 0.91^*^^*^	0.19 ± 0.12	1.12 ± 0.28^*^	0.09 ± 0.09	11.63 ± 1.97^*^^*^
WT	3.03 ± 0.34	0.70 ± 0.12	0.17 ± 0.09	0.63 ± 0.07	0.06 ± 0.03	0.32 ± 0.04	0.01 ± 0.01	4.91 ± 0.44

^*^ *P* < 0.05, ^*^^*^P < 0.01 indicate significant differences from the wild-type control.

^a^Mutation frequency was calculated as the number of mutation events divided by the length of the reference genome. ^b^Data for the wild-type control are from a previous study by Hase *et al*. [24].

ND = Not detected.

### Characteristics of SBSs, InDels and complex-type mutations

**Fig. 3.**
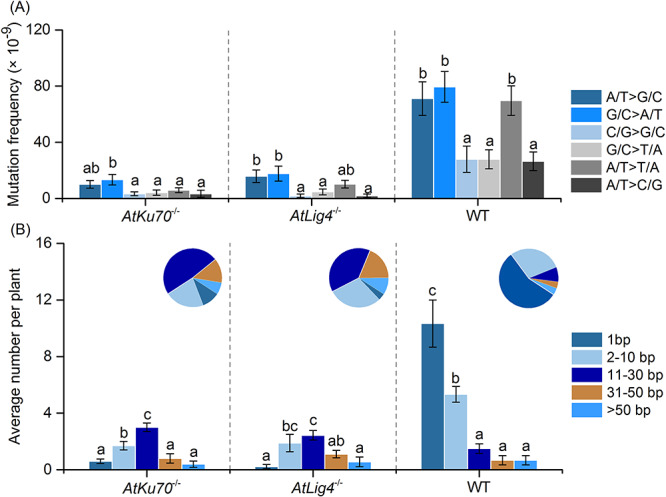
Characterization of SBS and InDel mutations. (**A**) Frequency and spectra of SBSs. (**B**) Length distribution of InDels. Data are presented as the mean ± standard error. Different letters indicate significant differences within each strain (*P* < 0.05). Data for the wild-type control are from a previous study by Hase *et al*. [24].

The SBS spectra revealed a lack of marked differences between the mutants and the wild-type control (Fig. 3A). A/T to G/C and G/C to A/T transitions and A/T to T/A transversions occurred slightly more frequently than the other SBSs in all strains. In the wild-type control, single-base deletions and insertions were the most common InDels, and the number of mutations decreased with increasing length (Fig. 3B). In contrast, in the NHEJ-deficient mutants, the most frequent InDels were 11–30 bp long, and the single-base InDel represented <10% of the total. These results imply that relatively long InDels are frequently induced in NHEJ-deficient mutants. As mentioned above, more complex-type mutations were detected per unit dose in the NHEJ-deficient mutants than in the wild-type control. Most of the complex-type mutations in the wild-type control comprised two or three SBSs (20 of 23, 87%), but these mutations were less abundant in the NHEJ-deficient mutants (4 out of 27, 15%), in which SBSs combined with short InDels were more frequent (Supplementary Table S5, see online supplementary material). Thus, the sequence change associated with complex-type mutations was more complicated in the NHEJ-deficient mutants than in the wild-type control.

### Sequence context of Del ≥2 bp

**Fig. 4.**
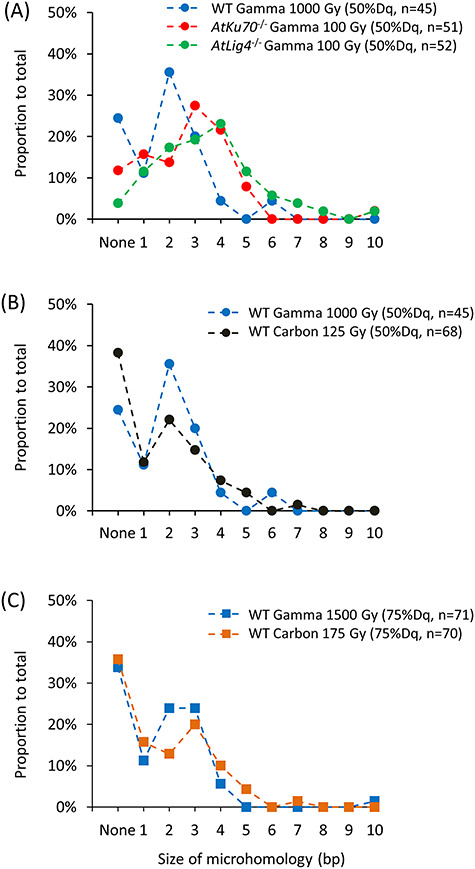
Distribution of the microhomology length at the rejoined site of Del ≥ 2bp. (A) Comparison between the wild-type control and NHEJ-deficient mutants at 50% Dq for gamma rays. (B, C) Comparison between gamma rays and carbon ions in the wild type at 50% Dq (B) and 75% Dq (C). Sequence details are provided in Supplementary Table S6. The wild-type data for the gamma irradiation are from a previous study by Hase *et al*. [24], whereas the data for the 17.3 MeV/u carbon ion irradiation (surface LET, 107 keV/μm) are from another study by Hase *et al*. [23]. The same wild-type data are shown in (**A**) and (**B**) for comparison.

An examination of the rejoined site of Del ≥2 bp mutations revealed that the most common microhomologies were 2 and 3–4 bp in the wild-type control and the NHEJ-deficient mutants, respectively (Fig. 4A and Supplementary Table S6, see online supplementary material). Additionally, the proportions of Del ≥2 bp mutations with no apparent microhomology were lower in the NHEJ-deficient mutants than in the wild-type control (i.e. 24, 12 and 4% in the wild-type, *AtKu70*^−/−^ and *AtLig4*^−/−^ plants, respectively). These results imply that, in the lack of *AtKu70* or *AtLig4*, the DNA ends are accessible to alternative pathways, requiring more microhomology.

### Transcription of DNA repair-related genes after gamma irradiation

**Fig. 5.**
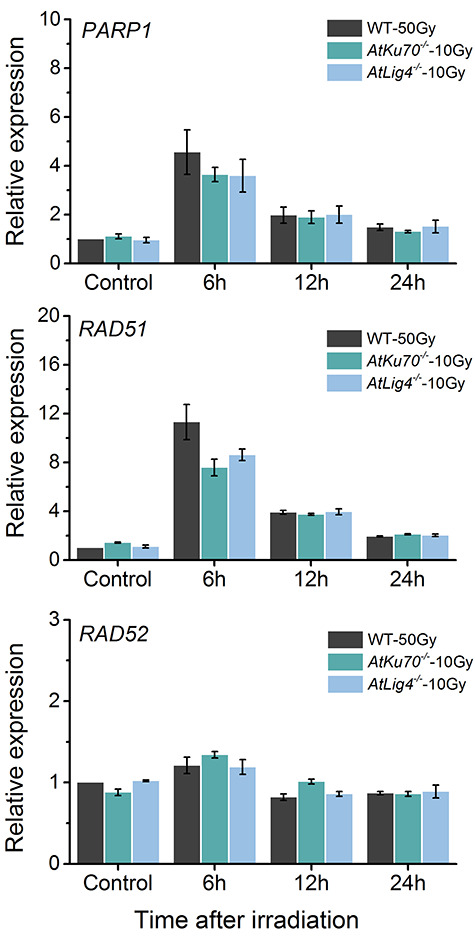
Transcription of DNA repair-related genes in response to gamma irradiation. One-week-old seedlings were irradiated and *PARP1*, *RAD51* and *RAD52* expression levels were examined at the indicated time-points. Data are presented as the mean ± standard error of three biological replications. Non-irradiated seedlings served as the control. *ACTIN2* was used as the endogenous control gene for the quantitative reverse-transcription PCR.

To assess whether the loss of *AtKu70* or *AtLig4* affects the transcription of genes involved in other DNA repair pathways, the expression levels of Arabidopsis homologs of *PARP1* (At2g31320, alt-EJ), *RAD51* (At5g20850, HR) and *RAD52–1* (At1g71310, SSA) were examined for up to 24 h after gamma irradiation. Specifically, 1-week-old *AtKu70*^−/−^ and *AtLig4*^−/−^ seedlings were exposed to 10 Gy, whereas the wild-type seedlings were treated with 50 Gy. All three genes were similarly expressed in the mutants and the wild-type control before irradiation (Fig. 5). Both *PARP1* and *RAD51* exhibited a clear transcriptional response at 6 h after irradiation, but the expression levels were generally similar between the mutants and the wild-type control over the 24-h period following irradiation. No clear transcriptional response was detected for *RAD52*. These results suggest that the loss of *AtKu70* or *AtLig4* does not substantially affect the transcription of *PARP1*, *RAD51* and *RAD52*.

## DISCUSSION

### Arabidopsis mutants lacking *AtKu70* and *AtLig4* are hypersensitive to gamma rays

The NHEJ-deficient Arabidopsis mutants were very sensitive to gamma rays, consistent with the notion that NHEJ is the major repair pathway in higher plants (Fig. 1). The fact the *AtKu70*^−/−^ mutant was more sensitive than the *AtLig4*^−/−^ mutant is also in accordance with previous research on Arabidopsis. The *AtKu70*^−/−^ and *AtKu80*^−/−^ mutants are reportedly more sensitive to gamma and Fe-ion irradiations than the *AtLig4*^−/−^ mutant based on the inhibition of root growth or the decrease in the number of developed leaves [25–27]. The observed difference in the radiation sensitivities of the *AtKu70*^−/−^ and *AtLig4*^−/−^ mutants may be because of a partial functional redundancy between AtLig4 and other ligases, or the Ku protein may affect other processes in addition to NHEJ. In rice, mutant calli with a homozygous T-DNA insertion in *OsKu70* exhibit severe growth retardation and cannot be used to regenerate plants, whereas homozygous *OsLig4* knockout plants remain viable [13, 28]. Ku heterodimer is required in protecting telomeres in Arabidopsis [29]. In mammalian cells, Ku70 interacts with DNA polymerase-β, a major component of base excision repair (BER), and the suppression of Ku70 decreases the efficiency of BER and increases sensitivity to DNA-damaging agents [30]. The opposite result was reported for a DT40 chicken B-lymphocyte cell line, in which a *lig4* mutant was more sensitive to X-rays than a *ku70* mutant [31]. Moreover, the *lig4* and *ku70* double mutant as well as the *ku70* single mutant exhibit similar radiation sensitivities, implying the Lig4 function cannot be substituted by the functions of other ligases in DT40. The situation may vary among species, possibly depending on the differences in DNA repair pathways. However, the available evidence supports the notion that the Ku protein has an important role beyond its function related to NHEJ in plants.

### Del ≥2 bp and complex-type are the main mutation types in NHEJ-deficient mutants

Because of the hypersensitivity of the NHEJ-deficient mutants to gamma rays, we were unable to irradiate them with the same dose as that used for the wild-type control in a previous study (1000 Gy corresponding to 50% *Dq*) [24]. Therefore, we characterized the mutations in the NHEJ-deficient mutants with 100 Gy as an equivalent dose. In tobacco BY-2 cells, the number of DSBs linearly increases with increasing doses of gamma rays and seven kinds of heavy ion beams with a LET of 9–440 keV/μm [32]. Assuming this is applicable to the irradiation of dry seeds, the DNA damage induced by gamma rays is ~10-fold more deleterious in the NHEJ-deficient background than in the wild-type background. The total number of mutation events per dose in the NHEJ-deficient mutants was more than twice that in the wild-type control (Table 1). The frequency of SBSs per dose was similar between the mutants and the wild-type control, whereas the frequencies of the induced Del ≥2 bp and complex-type mutations were about 7- and 4-fold higher in the mutants, respectively. Error-prone rejoining by the backup repair pathways likely led to the observed frequent induction of Del ≥2 bp and complex-type mutations. Our data uncovered longer deletions in the NHEJ-deficient mutants than in the wild-type control (Fig. 3B). In terms of the effects on gene function, deletion size is not very important, because single-base deletion is as severe as Del ≥2 bp. However, frequent induction of Del ≥2 bp and complex-type mutations suggest that the mutations induced in NHEJ-deficient mutants have a higher possibility to generate dysfunctional alleles than the wild-type control, in which SBS is the predominant type of mutation.

### Rejoining DNA ends in the NHEJ-deficient background requires increased microhomology

The mutants apparently required greater microhomology for rejoining the DNA ends (Fig. 4A). These features are suggestive of the involvement of alt-EJ and SSA, and are consistent with the observations in previous studies of NHEJ-deficient Arabidopsis and rice involving site-directed artificial nucleases [9–13]. Thus, when NHEJ is impaired, increased microhomology is required to repair the DNA damage caused by ionizing radiation, and the extensive DNA end resectioning results in the frequent induction of Del ≥2 bp. Although NHEJ is an error-prone repair pathway in plants, the results obtained in this study demonstrate that it can minimize the adverse effects of ionizing radiation more efficiently than the backup repair pathways.

High-LET radiation, such as heavy ion beams, induce more complicated and irreparable DNA damage than low-LET radiation [14, 15]. In fact, the DSBs induced by carbon ions are repaired considerably more slowly than those induced by X-rays [33]. In mammalian cells, NHEJ was suggested to be less efficient in repairing DNA damage induced by high-LET radiation than HR or alt-EJ [34, 35]. However, the relationship between radiation quality and the use of different DNA repair pathways remains unclear in higher plants. When we reanalyzed the previously obtained data for gamma and carbon-ion irradiation in wild-type Arabidopsis, 2 bp was the most frequent microhomology for both irradiations at 50% *Dq* (Fig. 4B). The corresponding data for 75% *Dq* also revealed a similar distribution between gamma and carbon-ion irradiation (Fig. 4C). Although carbon ions induce larger deletions than gamma rays on average [24], these results suggest that the rejoining of broken DNA ends due to carbon ions and gamma rays likely occurs similarly. Interestingly, the 2-bp peaks observed at 50% *Dq* were less evident at 75% *Dq*. The microhomology length might increase with increasing doses, although it was shorter than that observed in the NHEJ-deficient mutants. Both alt-EJ and SSA might be used to a greater extent at relatively high radiation doses, irrespective of the radiation quality.

### NHEJ suppresses the generation of complicated mutations

The frequency and complexity of the complex-type mutation increased in the *AtKu70*^−/−^ and *AtLig4*^−/−^ mutants (Table 1, Fig. 2 and Supplementary Table S5). Complex-type mutations comprising short InDels were rare in the gamma- and carbon-ion-irradiated wild-type control (3 of 23 and 1 of 14, respectively; Supplementary Table S5), whereas they were the major mutations in the mutants (23 of 27 in total). Furthermore, in earlier investigations, complex-type mutations comprising short InDels were infrequent among the mutations induced by site-directed nucleases in NHEJ-deficient mutants [9, 10, 12, 13]. Therefore, these kinds of mutations are unique to the radiation-induced DNA damage repaired by the Ku-independent pathway. This also suggests that more than two SBSs that are consecutive or adjacent to each other and complex-type mutations comprising short InDels are generated by different repair pathways. Unfortunately, determining the existence and length of microhomologies as we did for simple deletions is difficult for complex-type mutations. Therefore, we cannot discuss the mechanism generating the mutation in detail. Nevertheless, it is plausible that these kinds of mutations are induced during the processing of DNA ends generated in close proximity. These considerations suggest that NHEJ is the main pathway for rejoining the broken DNA ends due to ionizing radiation and is more precise than the alt-EJ and SSA pathways.

### NHEJ deficiency may not affect the transcription of genes involved in other DNA repair pathways

Our results suggest the involvement of alt-EJ and/or SSA for repairing radiation-induced DNA damage in an NHEJ-deficient background. However, relatively little information is available for assessing whether NHEJ deficiency affects the transcription of genes involved in other repair pathways. The expression levels of Arabidopsis *PARP1* and *RAD51* genes quickly increase in response to gamma rays in dose- and dose rate-dependent manners [36]. Additionally, *PARP1* and *RAD51* are responsive to DSBs generated by bleomycin, but *RAD52* is not [37, 38]. The expression levels of three genes in 50 Gy-irradiated wild-type seedlings were consistent with the above results (Fig. 5). Regarding the wild-type seedling survival rate, 50 Gy corresponds to ~32% *Dq* (155 Gy, data not shown). The mutant seedlings were irradiated with 10 Gy because they were hypersensitive. We were unable to detect any major differences between the wild-type control and the NHEJ-deficient mutants at the tested irradiation doses. Accordingly, a deficiency in NHEJ may not influence the transcription of genes involved in other DNA repair pathways.

## CONCLUSION

The results presented herein indicate that NHEJ is a very important DNA repair pathway for minimizing the deleterious effects of ionizing radiations because it prevents extensive DNA end resectioning and is more precise then the backup pathways in higher plants.

## Supplementary Material

Supplementary_materials_rraa059

Supplementary_Table_S5_rraa059

Supplementary_Table_S6_rraa059
