# Supplementary material for: Characterization of gamma irradiation-induced mutations in Arabidopsis mutants deficient in non-homologous end joining
Source: J Radiat Res. 2020 Aug 7;61(5):639–47. doi: 10.1093/jrr/rraa059 (PMC7482170; doi:10.1093/jrr/rraa059)
Supplement: Supplementary_materials_rraa059 [file supplementary_materials_rraa059.docx]

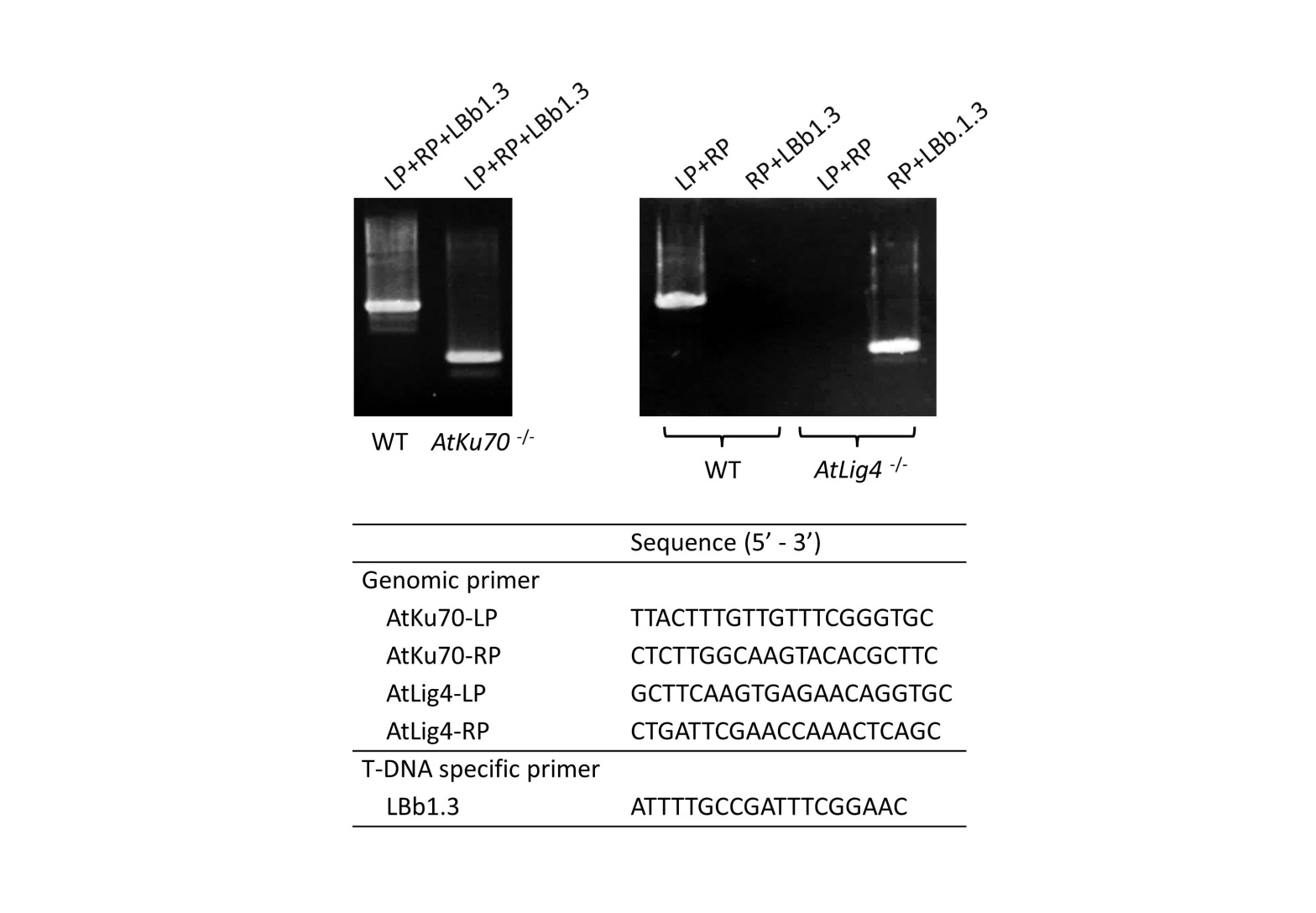


**Supplementary Figure S1.** Identification of homozygous lines for the T-DNA insertion at the *AtKu70* and *AtLig4* loci.


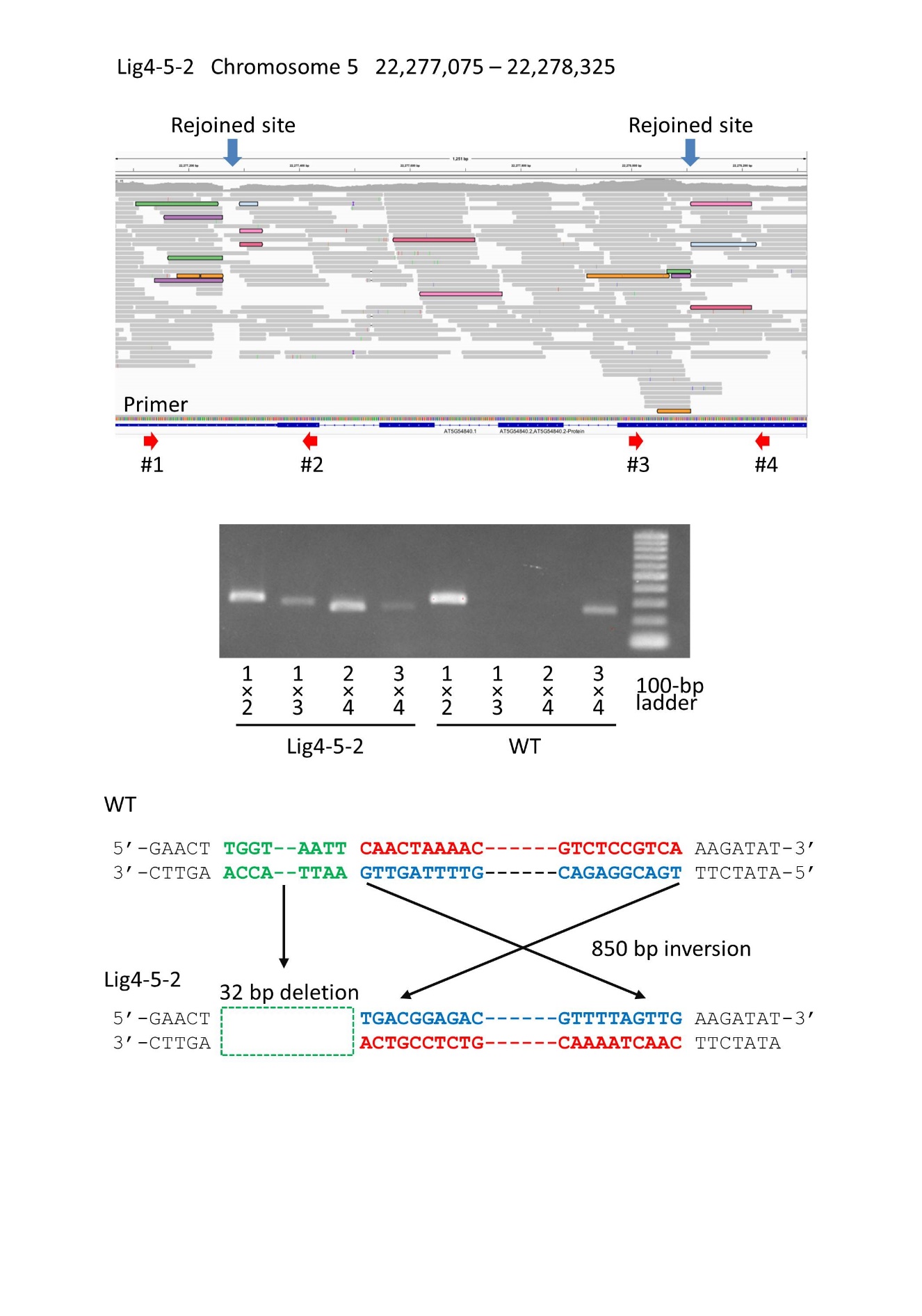


**Supplementary Figure S2.** Verification of the heterozygous inversion detected in lig4-5-2. (Top) Several pairs of sequence reads, which are highlighted in different colors in a snapshot of the IGV browser, suggest the occurrence of heterozygous inversion. The red arrows indicate the position and direction of PCR primers. (Middle) PCR products amplified with the indicated primer pairs. (Bottom) Sequence alteration confirmed by Sanger sequencing. Details regarding the primer sequences are provided in Supplementary Table S2.

| **Supplementary Table S1.** Summary of mapping results | | | |
| --- | --- | --- | --- |
| Sample | Total mapped base | Mean depth  of coverage (×) | % bases  above 10× |
| lig4-1-6 | 7473097473 | 62.6 | 99.9 |
| lig4-10-1 | 2984232067 | 25.0 | 97.8 |
| lig4-2-3 | 7006717727 | 58.6 | 99.9 |
| lig4-3-5 | 3850597793 | 32.2 | 99.6 |
| lig4-4-4 | 4359625969 | 36.5 | 99.7 |
| lig4-5-2 | 4416855648 | 37.0 | 99.8 |
| lig4-6-2 | 4292720104 | 35.9 | 99.7 |
| lig4-7-4 | 4030300662 | 33.7 | 99.7 |
| lig4-9-3 | 8979834686 | 75.2 | 100 |
| ku70-1-3 | 5013508749 | 42.0 | 99.9 |
| ku70-10-1 | 3063439413 | 25.6 | 98.0 |
| ku70-2-4 | 2812394209 | 23.5 | 96.8 |
| ku70-3-1 | 4798537971 | 40.2 | 99.9 |
| ku70-4-1 | 4507131413 | 37.7 | 99.8 |
| ku70-5-1 | 4689283806 | 39.3 | 99.9 |
| ku70-6-1 | 3567888126 | 29.9 | 99.4 |
| ku70-7-1 | 3562890184 | 29.8 | 99.5 |
| ku70-8-1 | 2931017364 | 24.5 | 97.7 |
| ku70-9-1 | 3778144762 | 31.6 | 99.5 |

| **Supplementary Table S2.** Verification of some detected mutations by Sanger sequencing | | | | | | | |
| --- | --- | --- | --- | --- | --- | --- | --- |
| Number | Sample | Chr | Position | Type of mutation | Zygosity | Forward and reverse primer (5’-3’) | Result |
| 1 | lig4-5-2 | 1 | 11929635 | –1 | Hetero | F: GAACCAAACCAAAAGCGAGA  R: TCATTGAACCGGAAGTGTGA | True |
| 2 | ku70-9-1 | 1 | 18792818 | –1 | Homo | F: ATCACGGGACGGGTAAAAA  R: CGACTAAACTAACCGGCAGTG | True |
| 3 | ku70-10-1 | 1 | 21671258 | –1 | Hetero | F: GTAGCGGTTGGTGTTCGATT  R: TTAGTGGATCCGCTCATGCT | True |
| 4 | ku70-6-1 | 2 | 2327017 | +1 | Hetero | F: GTGGGGTTCTTCTGCTTTCA  R: CGACATCAACCTTTCGATCA | True |
| 5 | ku70-8-1 | 3 | 15354344 | +1 | Hetero | F: CCCTAGTCATTCGGATTTGG  R: TGGTAGAGGGGTCGAATAGG | True |
| 6 | lig4-2-3 | 5 | 25065565 | Del ≥2bp (258 bp) | Homo | F: GGAGATGGGTTTGTGCAGAT  R: TTCCATTCCTCCAAATGGTC | True |
| 7 | lig4-2-3 | 5 | 7220895 | Del ≥2bp (200 bp) | Homo | F: TGTGGACGCTGTTTTCAAAG  R: TGGTGAGGTTCGAGTTTATCG | True |
| 8 | lig4-4-4 | 5 | 15052920 | Del ≥2bp (126 bp) | Hetero | F: GTGGCAACTGCCTCCTTCT  R: AGCCGGCAATAGCAAATAGA | True |
| 9 | ku70-5-1 | 4 | 16950930 | Del ≥2bp (75 bp) | Homo | F: CCATATGAGTGTGGGTCACG  R: AAGCAAATATGGGCCACAAG | True |
| 10 | lig4-4-4 | 3 | 21514441 | Del ≥2bp (39 bp) | Homo | F: GTTTGTTTGGAAACGCGAAT  R: GGAATCGACTTGAGGGATCA | True |
| 11 | ku70-6-1 | 3 | 13331178 | Del ≥2bp (22 bp) | Hetero | F: GGTTTCCAGAAACACCACTTG  R: TCCCACTATTGAAACACTCGTT | True |
| 12 | lig4-7-4 | 2 | 17457016 | Del ≥2bp (21 bp) | Hetero | F: ATCACATCCCGAGGAATCAA  R: CAGTGTATGAGCTGGCCAAA | True |
| 13 | ku70-7-1 | 3 | 3377589 | Del ≥2bp (18 bp) | Hetero | F: CTTTGAAGGAATGCCTCTGC  R: ATCCGATATCGTCATGCACA | True |
| 14 | ku70-4-1 | 3 | 14707408 | Del ≥2bp (12 bp) | Hetero | F: ATCTGGTCCGAAATGCTGTC  R: CCTTAATTGTCGTGGGAGGA | True |
| 15 | lig4-1-6 | 1 | 16659690 | Del ≥2bp (11 bp) | Homo | F: TGGACTCATGCTCAGAAACG  R: CACACTTACACGGGACACCA | True |
| 16 | ku70-4-1 | 1 | 1589606 | Del ≥2bp (6 bp) | Hetero | F: CGTAGGCGCTTTTTCATGTT  R: CATCGTGCTTTGCTGTGAAT | True |
| 17 | ku70-7-1 | 1 | 2200180 | Ins ≥2bp (6 bp) | Homo | F: AAAAAGCCCATCAGGAAAGC  R: GAAACATGAACACGGAACGA | True |
| 18 | lig4-6-2 | 2 | 1032360 | Ins ≥2bp (11 bp) | Homo | F: CACGAAACACATCGATCACC  R: CCAAACATTGGGCCTTTATG | True |
| 19 | ku70-9-1 | 3 | 824772 | Ins ≥2bp (22 bp) | Homo | F: GCCATGAAAGATGGAAAGGA  R: AGCCCCACTAAGTCCAATCA | True |
| 20 | lig4-2-3 | 4 | 16663196 | Complex | Homo | F: GAGGTTGTAAAGGGCATCCA  R: CGTGAGGTTTCACCGATTGT | True |
| 21 | lig4-7-4 | 5 | 16414599 | Complex | Hetero | F: TGAGCTCTCTCCCTCTCAGG  R: GGCTCATAGGGTCTCAAGCA | True |
| 22 | ku70-6-1 | 1 | 461315 | Complex | Homo | F: CCCATTAAAACGGTTTCAGAC  R: GGACCCGGGAAGAGTTAAAA | True |
| 23 | lig4-2-3 | 3 | 1290981 | Complex | Hetero | F: AATGGCGTCATTTTCCTGAG  R: AACGAACGAATCGAATCCAC | True |
| 24 | lig4-4-4 | 4 | 16290515 | Complex | Homo | F: TCGTTGTCTTTGCAGGAGTG  R: ACCTGAGCATGTCGGGTATC | True |
| 25 | lig4-3-5 | 1 | 687482 | Complex | Homo | F: TCGAAACGACAGAGGAGGTT  R: GCGTTTTTGTTTTCCACACG | True |
| 26 | ku70-3-1 | 5 | 3909383 | Complex | Homo | F: AATGAATCGCAGCCATTTTC  R: GTTCGCAGGAAGGTGAATGT | True |
| 27 | ku70-4-1 | 5 | 8355730 | Complex | Hetero | F: AGGGATTACCGGAGCAAGAT  R: CAGCACAAAACAATGAACTCG | True |
| 28 | lig4-3-5 | 1 | 16273789 | SBS (A to G) | Hetero | F: AATCCCCTTCGACCACAAAT  R: CAACCAATTGCATCCTAACAAG | True |
| 29 | ku70-3-1 | 1 | 21652470 | SBS (G to A) | Homo | F: GGACCGACCTTAGTTGGTTTT  R: GCGAATTCACAGTCGTCCTA | True |
| 30 | lig4-4-4 | 4 | 4267494 | SBS (C to T) | Homo | F: CGCCTTAACAAGCAAAGAGG  R: AGATCAAGCCTCGGTCAAGA | True |
| 31 | lig4-2-3 | 4 | 11389854 | SBS (T to C) | Hetero | F: GCAAGACCCCAAAACTGAAA  R: GCAGACAAATCCCGAAACTC | True |
| 32 | lig4-6-2 | 4 | 15831580 | SBS (C to T) | Hetero | F: AAAAGGAACGGATTGTGGTG  R: ACTGGGTGGGGATAGTTGTG | True |
| 33 | lig4-2-3 | 5 | 15745648 | SBS (C to T) | Hetero | F: AAGTCGCAGAGTGGTTGCTT  R: GTTTGGGCTTTGTTTTCAGC | True |
| 34 | lig4-5-2 | 5 | 22277262 | SV  (Details are shown in Supplementary Figure S2) | Hetero | 1: AAGAAAAGTCGACGTGCTAAAT  2: TAGCCAGGCAAGAAAATGGA  3: ACAAAAGTAGTCTTCCCTGTTTGA  4: TGGAGATTTCTTTAGGTTTCTATGG | True |

| **Supplementary Table S3.** Primers used for quantitative reverse-transcription PCR | | |
| --- | --- | --- |
|  | Forward primer (5’-3’) | Reverse primer (5’-3’) |
| *PARP1* | TGGAGCAGGGAGTAACACAA | CCTTCAGGCTTGGAGAATATACG |
| *RAD51* | TGATGCCAAGGTTGACAAGATT | CAGAGCGGAACTCACCATATAAC |
| *RAD52* | GGGTGCTCCATCAAATACATCC | CACACGATAAGCCACAGTAACA |
| *ACTIN2* | TGTGCCAATCTACGAGGGTTT | TTTCCCGCTCTGCTGTTGT |

| **Supplementary Table S4.** Number and zygosity of mutations | | | | | | | | |  |
| --- | --- | --- | --- | --- | --- | --- | --- | --- | --- |
|  | *AtKu70*^-/-^ | |  | *AtLig4*^-/-^ | |  | WT | |  |
|  | Hetero | Homo |  | Hetero | Homo |  | Hetero | Homo |  |
| SBS | 39 | 9 |  | 42 | 14 |  | 159 | 58 |  |
| –1 | 3 | 1 |  | 2 | 0 |  | 33 | 17 |  |
| +1 | 1 | 1 |  | 0 | 0 |  | 8 | 4 |  |
| Del ≥2bp | 37 | 14 |  | 35 | 17 |  | 33 | 12 |  |
| Ins ≥2bp | 3 | 3 |  | 1 | 1 |  | 2 | 2 |  |
| Complex | 10 | 7 |  | 9 | 3 |  | 18 | 5 |  |
| SV | 0 | 0 |  | 1 | 0 |  | 1 | 0 |  |
| Total | 93 | 35 |  | 90 | 35 |  | 254 | 98 |  |
| Data for the wild-type control are from a previous study by Hase et al. [24]. | | | | | | | | | |
