# Supplementary material for: Characterization of gamma irradiation-induced mutations in Arabidopsis mutants deficient in non-homologous end joining
Source: J Radiat Res. 2020 Aug 7;61(5):639–47. doi: 10.1093/jrr/rraa059 (PMC7482170; doi:10.1093/jrr/rraa059)
Supplement: Supplementary_Table_S5_rraa059 [file supplementary_table_s5_rraa059.pdf]

Supplementary Table S5

Complex type induced by 100 Gy of gamma rays in *atku70* mutant.

Sample	Chr#	Position	Zygosity	Original sequence	Altered sequence	Detail of mutation
ku70-3-1	5	7,026,319	homo	ggttggacCggAgtatgacg	ggttggacGggGgtatgacg	SBSx2
ku70-4-1	5	24,463,417	hetero	tgtattcaCCgcaatgac	tgtattcaATgcaatgac	SBSx2
ku70-8-1	1	17,283,264	hetero	ggtgagatTcaAGCATTATttAccatcttg	ggtgagatAcattCccatcttg	SBS, -8, SBS
ku70-4-1	3	19,734,466	hetero	cccaagtcAtaTctTTttctCGTGttGcTGattctaa	cccaagtcGtCTTAGGaGctCCttCACAGtctttAcATattctaa	SBS, +6, SBSx3, +5, -4, SBSx3
ku70-9-1	5	7,328,692	homo	aactcccgCAAACGCACctAttatcaaa	aactcccgctGGTttatcaaa	-9, +2(-1+3)
ku70-6-1	1	461,315	homo	atgtacgaTTAATttAtTttcttagg	atgtacgattGtGttcttagg	-5, SBSx2
ku70-7-1	1	23,399,727	homo	ctctcattAAGCTATC---ATTGCATCaaacatagctgaatagtactt	ctctcattaaacatagctgaaTAGTACTTtagtactt	-35, +8
ku70-7-1	5	5,697,912	hetero	cacccatcTGTaaaaaattaaaCAGttgaGTGtcCGCAtataaattt	cacccatcaaaaaattaaattgatctataaattt	-3, -3, -3, -4
ku70-10-1	1	23,442,443	homo	tgtgatatAAGaaattatCaatagtaatt	tgtgatataaatCCtatTaaTAACtagtaatt	-3, +2, SBS, +4
ku70-2-4	1	11,170,815	hetero	atctaagaCCaaGtcCATCGATAGTTTTtagtgggt	atctaagaaaAtcTAtagtgggt	-2, SBS, -11(-13+2)
ku70-1-3	3	500,878	hetero	taggatgtGACGAATGAAAActCAAACAACAACAAAAAAAACACaaaactca	taggatgtctATaaaactca	-12, -20(-22+2)
ku70-1-3	5	14,008,889	hetero	gagaaacaaGgttAggatgatt	gagaaacaGCCAATaAgtCTTTCtggatgatt	+6, SBS, +5, -1
ku70-10-1	5	21,426,077	homo	ggattgtcGGCgaCACTttGttTAaatcggta	ggattgtcCATATGAgattTttGGaatcggta	+4(-3+7), -4, SBSx3
ku70-4-1	5	8,355,746	hetero	atctcaaaacaaaGcttttcacaaATTTTATCAATCTTgcttttTGATACATGAATAAtcagagag	atctcaaaAGAAAAGATACacaaaTcttttcacaagcttttcagagag	+11, SBS, -14, -14
ku70-2-4	1	22,889,111	hetero	tttccaaatttCcctttctt	tttccaaaTtttAcctttctt	+1, SBS

Complex type induced by 100 Gy of gamma rays in *atlig4* mutant.

Sample	Chr#	Position	Zygosity	Original sequence	Altered sequence	Detail of mutation
lig4-9-3	4	17,517,268	hetero	agaatgttCGTAaacCCCCTCAA---TGCTTCCTttgattcc	agaatgttTTGGaacttgattcc	SBSx4, -22
lig4-4-4	4	12,539,016	hetero	caagcagaGagCtcaaacggg	caagcagaAagTtGGACcaaacggg	SBSx2, +4
lig4-2-3	3	6,857,729	hetero	tctttttaAcaTaAttattact	tctttttaGcaGaGTTttattact	SBSx2, +2(-3+2)
lig4-3-5	1	687,482	homo	cgtcatttCAgtcttcaa	cgtcatttTTgtcttcaa	SBSx2
lig4-6-2	3	18,478,665	hetero	ggatagagAAagagatag	ggatagagTTagagatag	SBSx2
lig4-7-4	5	16,414,599	homo	gaagcagaGatTAGTGAAAAATGTgggaacaa	gaagcagaTatGgggaacaa	SBS, -12(-13+1)
lig4-10-1	3	18,013,825	hetero	tccaacacTaaaAcgacgacg	tccaacacGaaAGaTcgacgacg	SBS, +2, SBS
lig4-2-3	4	16,663,196	hetero	aatcaaaaGTTTGGAGGATTaatctAGaaagacac	aatcaaaaaaATTCCTCTtctTCaaagacac	-12, +8, SBSx2
lig4-7-4	4	11,796,517	homo	tttttcctTAAGAGAATAatataAAACAAGA---AGCTTATTccataact	tttttcctatataccataact	-10, -32
lig4-7-4	4	4,782,131	hetero	actacaaaccTatTATCATCTACCGTCCAGatgtcctc	actacaaaGATGccAatatgtcctc	+4, SBS, -17
lig4-6-2	3	10,102,461	hetero	acacgcacAAAAGCaatCAgAaacaaatg	acacgcacCATTCTCAaatTGgCaacaaatg	+2(-6+8), SBSx3
lig4-4-4	1	16,453,396	hetero	acaccataCtgAaTtaAgttacacc	acaccataTTACACATGATTTtgTaAtaTgttacacc	+12(-1+13), SBSx3

Complex type induced by 1,000 Gy of gamma rays in the wild type.

Sample	Chr#	Position	Zygosity	Original sequence	Altered sequence	Detail of mutation
1000Gy-2	3	10,638,188	hetero	aactCGAcaat	aactATTcaat	SBSx3
1000Gy-4	1	6,107,287	hetero	ggttCTttgc	ggttAAttgc	SBSx2
1000Gy-4	1	15,679,388	hetero	agctGAaatg	agctTCaatg	SBSx2
1000Gy-5	1	18,863,822	hetero	ctctTTtttc	ctctAAtttc	SBSx2
1000Gy-1	1	20,139,035	hetero	gattGGagga	gattTTagga	SBSx2
1000Gy-5	1	22,598,127	hetero	tatgTCtgcg	tatgAGtgcg	SBSx2
1000Gy-2	2	6,369,167	homo	ttgaAGtgtt	ttgaGTtgtt	SBSx2
1000Gy-6	2	18,699,983	hetero	ttcaTCgagg	ttcaATgagg	SBSx2
1000Gy-5	3	394,161	homo	gcatGCgaaa	gcatAAgaaa	SBSx2
1000Gy-6	3	3,441,297	homo	aattCtGcata	aattAtTcata	SBSx2
1000Gy-2	3	5,794,486	hetero	ttttGGtttt	ttttATtttt	SBSx2
1000Gy-1	3	12,355,708	homo	ttttGGagag	ttttAAagag	SBSx2
1000Gy-2	3	20,650,319	hetero	gaacTCacat	gaacATacat	SBSx2
1000Gy-3	4	3,931,349	hetero	aagaACctcc	aagaCTctcc	SBSx2
1000Gy-5	4	6,052,447	hetero	gatcTCtgaa	gatcATtgaa	SBSx2
1000Gy-3	4	7,087,397	hetero	tttcTCatat	tttcATatat	SBSx2
1000Gy-4	4	14,495,837	hetero	ttccTgCaatc	ttccAgAaatc	SBSx2
1000Gy-4	4	15,070,705	hetero	agaaTAagta	agaaATagta	SBSx2
1000Gy-3	5	574,166	hetero	tggtCAaatg	tggtGTaatg	SBSx2
1000Gy-4	5	19,855,998	hetero	atgaTAgacg	atgaCTgacg	SBSx2
1000Gy-1	2	10,076,397	hetero	tcgtTActAtaac	tcgtctGtaac	-2, SBS
1000Gy-1	5	13,744,603	hetero	tagagcgttAggag	tagaTgcgttGggag	+1, SBS
1000Gy-2	5	21,439,863	homo	actattCcttt	actaTttGcttt	+1, SBS

Complex type induced by 125 Gy of carbon ions in the wild type.

Sample	Chr#	Position	Zygosity	Original sequence	Altered sequence	Detail of mutation
125-10-5	2	5,641,286	hetero	tattCCattt	tattATattt	SBSx2
125-12-1	2	13,864,099	hetero	caccAccAccgt	caccCccCccgt	SBSx2
125-12-1	5	1,553,890	homo	gaaaAGtaaa	gaaaTTtaaa	SBSx2
125-2-2	1	24,907,911	hetero	aaatTacggAttta	aaatCacggGttta	SBSx2
125-2-2	4	6,671,908	homo	tgggGTcgtg	tgggAAcgtg	SBSx2
125-2-2	4	11,005,321	homo	tgctGTcaaa	tgctAAcaaa	SBSx2
125-2-2	5	21,587,693	hetero	ttatCTtcat	ttatTCtcat	SBSx2
125-4-1	2	2,827,384	hetero	ttggAGagac	ttggGAagac	SBSx2
125-4-1	5	16,591,716	hetero	gctaAtTtcgt	gctaTtCtcgt	SBSx2
125-5-1	5	14,691,336	homo	gcaaTaaaGattc	gcaaAaaaCattc	SBSx2
125-5-1	5	20,905,119	hetero	ataaGGattt	ataaAAattt	SBSx2
125-5-1	5	22,060,358	hetero	ctaaCCaaaa	ctaaAAaaaa	SBSx2
125-6-1	3	15,886,369	hetero	attgCaCatat	attgAaTatat	SBSx2
125-5-1	4	10,605,193	homo	taaaAAGGTGAAacTaaaa	taaaTTCacCaaaa	-5(-8+3), SBS
