# Supplementary material for: Characterization of gamma irradiation-induced mutations in Arabidopsis mutants deficient in non-homologous end joining
Source: J Radiat Res. 2020 Aug 7;61(5):639–47. doi: 10.1093/jrr/rraa059 (PMC7482170; doi:10.1093/jrr/rraa059)
Supplement: Supplementary_Table_S6_rraa059 [file supplementary_table_s6_rraa059.pdf]

Supplementary Table S6

Deletion >=2bp induced by 100 Gy of gamma rays in *atku70* mutant.

Sample	Chr#	Position	Zygosity	Deletion size	Original sequence	Altered sequence	Size of MH
ku70-2-4	5	12,379,636	homo	-5	ccttcaacCGAATctatgtga	ccttcaacctatgtga	1
ku70-2-4	5	18,550,182	hetero	-10	aaaaaaataTGATATTATTtaaattaa	aaaaaatataaattaa	1
ku70-3-1	2	3,121,111	hetero	-16	actagtgtCGATCGACACCTGATActtcatag	actagtgtcttcatag	1
ku70-6-1	5	16,389,305	hetero	-19	tccattatGTTTTTATATAGGGTTCAGgatttaaa	tccattatgatttaaa	1
ku70-8-1	1	825,504	hetero	-23	gtgattagTAATGTTC---TACATATCttaacagt	gtgattagttaacagt	1
ku70-8-1	4	11,468,390	hetero	-23	caccaagcTCATCAAG---AGCCAAAAtaacaagg	caccaagctaacaagg	1
ku70-6-1	4	15,330,946	hetero	-38	tactgtttCTATTATT---AGGCCAAAccaaaaga	tactgtttccaaaaga	1
ku70-5-1	4	10,351,976	hetero	-45	tggagatgCCTTTAAG---AGGAGTCAcgagcaat	tggagatgcgagcaat	1
ku70-5-1	1	21,152,190	hetero	-2	tacacgtgTAtatatata	tacacgtgtatatata	2
ku70-8-1	1	4,781,377	hetero	-14	acaaaccaATGTCTCTAGTTTTatatcaag	acaaaccaatatcaag	2
ku70-2-4	2	313,975	homo	-16	tattaattTTTCTATTTTGAAACAttagctaa	tattaattttagctaa	2
ku70-9-1	1	13,114,909	homo	-17	attgcggaAAATTGTTTATATAGGTaatatgta	attgcggaaatatgta	2
ku70-5-1	5	7,749,849	homo	-18	ctcctgagCATGTTCTCAAGGCATTAcaggttct	ctcctgagcaggttct	2
ku70-9-1	1	19,980,596	hetero	-18	tcgaaggtTTCTTGACGCCGTCAGGAttggcaaa	tcgaaggtttggcaaa	2
ku70-7-1	5	7,316,417	hetero	-28	tcacaaacCTCTCAAC---TCGCTTTTctgcacaa	tcacaaacctgcacaa	2
ku70-5-1	1	20,267,039	homo	-51(-54+3)	tggcaacgCGGTGGAA---TATACCTGgtggtcga	tggcaacgCTCgtggtcga	3
ku70-10-1	1	6,067,447	homo	-12(-13+1)	ccatttccTTTGCCACTACTTgagccgtt	ccatttccAgagccgtt	3
ku70-9-1	1	3,180,159	hetero	-8	tcaatggcAACCAAGTaacgctgc	tcaatggcaacgctgc	3
ku70-5-1	1	11,168,765	hetero	-11	tggctaggTTTTAAGGCTAtttcgttg	tggctaggtttcgttg	3
ku70-1-3	3	11,283,160	homo	-13	atcggtcaTTCTCTGTATTTCttcgaagg	atcggtcattcgaagg	3
ku70-8-1	3	7,841,958	hetero	-13	cctcccagTACTTTGACCCACtacgacat	cctcccagtacgacat	3
ku70-9-1	5	1,366,403	hetero	-20	acatatcaTTATCAAGTAGTTAAAAGACttaccaag	acatatcattaccaag	3
ku70-6-1	3	13,331,178	hetero	-22	ttcaaaatATATTTCA---TTGAGAAGatacttgt	ttcaaaatatacttgt	3
ku70-4-1	1	10,270,121	hetero	-24	gagattacTAAGCTAC---CAAAAATAtaaaaagc	gagattactaaaaagc	3
ku70-5-1	4	7,172,241	hetero	-25	ctacaataATGTTGTG---AGAAGTTTatgggaaa	ctacaataatgggaaa	3
ku70-6-1	1	15,761,970	hetero	-28	caatctcaGCAGATGT---GCCATTGGgcacctca	caatctcagcacctca	3
ku70-3-1	3	12,121,181	hetero	-30	aacttcttATAACTCG---ACTCAACCataccgtt	aacttcttataccgtt	3
ku70-6-1	2	2,482,895	hetero	-40	aaatggtaATGCTAAA---ATATGCTTatgtcttg	aaatggtaatgtcttg	3
ku70-5-1	4	16,950,930	hetero	-75	tatttgttGTATAACA---AGCAATACgtacgagg	tatttgttgtacgagg	3
ku70-3-1	2	501,647	hetero	-4	aatgttacTATAtataaaat	aatgttactataaaat	4
ku70-1-3	3	10,415,480	hetero	-5	aacctatgACCACaccataat	aacctatgaccataat	4
ku70-4-1	1	1,589,606	hetero	-6	taataataAAATAGaaatttc	taataataaaatttc	4
ku70-1-3	2	16,504,929	hetero	-9	aaaatttaCAACCGGTTtaatccga	aaaatttataatccga	4
ku70-10-1	2	4,517,260	homo	-10	cttcttatGCGGCCAGCAccgcggtc	cttcttatccgcggtc	4
ku70-4-1	3	14,707,408	hetero	-12	cctttctcAAGTAACTCTATaagttgct	cctttctcaagttgct	4
ku70-2-4	1	9,736,535	homo	-23	aactttcaGAATTGTT---ATCATTCTgaatatgt	aactttcagaatatgt	4
ku70-1-3	5	17,174,303	hetero	-27	ctcatttaTTTGATTA---GTTTTTATtttgtaat	ctcatttatttgtaat	4
ku70-2-4	2	3,699,248	homo	-32	gctcttcaCAAGATTA---GATTTTTCcaagttcc	gctcttcacaagttcc	4
ku70-10-1	3	6,291,516	homo	-38	ccattcaaACTACGGG---CACAGGTGcaaatttc	ccattcaacaaatttc	4
ku70-10-1	5	17,813,636	hetero	-60	aacgtaacTTAAGTGA---CATATGATttaaaggt	aacgtaacttaaaggt	4
ku70-10-1	3	11,428,334	homo	-16	aggaagaaGATTTATCAAGCTTTGgatttcct	aggaagaagatttcct	5
ku70-5-1	1	13,708,322	hetero	-22	actcaactTGGACATT---ACAAAGAGtggactag	actcaacttggactag	5
ku70-2-4	4	17,020,191	homo	-31	cctgtttaAGTTTGCA---TTTTTTGCagttttac	cctgtttaagttttac	5
ku70-3-1	4	13,717,811	hetero	-48	gtgactatTCTTGAGT---GACGATAAtcttgtga	gtgactatAtcttgtga	5
ku70-9-1	3	4,987,599	hetero	-10	ccccATATATATATatatatatatatat	ccccatatatatatatat	10
ku70-5-1	4	6,267,122	hetero	-4(-7+4)	cggtcgaaAATGAAGttgttttg	cggtcgaaATCttgttttg	none
ku70-2-4	2	7,258,156	hetero	-36(-42+6)	gtgtaaaaGAAAAATA---CCAAACATaaaataaa	gtgtaaaaCATTAAaaaataaa	none
ku70-1-3	3	1,800,782	hetero	-10(-11+1)	agagctgtACGTAATAAGAggatgttg	agagctgtTgatgttg	none
ku70-4-1	1	19,824,923	hetero	-15	ataatctaGTAGAATAGGAAGACtccaaggc	ataatctatccaaggc	none
ku70-4-1	3	3,125,674	hetero	-18	atttattaAACTAACGAGCATTATTGtcaggtgt	atttattatcaggtgt	none
ku70-9-1	3	13,610,991	homo	-100	ataccaccCACAGTCG---TGTTTTCCtgggcaga	ataccacctgggcaga	none

Deletion >=2bp induced by 100 Gy of gamma rays in *atlig4* mutant.

Sample	Chr#	Position	Zygosity	Deletion size	Original sequence	Altered sequence	Size of MH
lig4-2-3	3	13,130,226	hetero	-11	ttaacaacAAAGAAATCATatcaaaaa	ttaacaacatcaaaaa	1
lig4-7-4	3	9,866,578	homo	-11	caccatttTGAATGAAACCtaaattaa	caccattttaaattaa	1
lig4-3-5	1	12,084,717	homo	-21	agataaatGAATTTTG---CAATAAAAgtttgtta	agataaatgtttgtta	1
lig4-7-4	2	17,457,016	homo	-21	cgtttcaaCGGCCACG---GGTTCCACcttctgcc	cgtttcaacttctgcc	1
lig4-6-2	5	349,515	hetero	-24	ttttttctTCTTTCCT---ATGTATGAttaatata	ttttttcttaatata	1
lig4-3-5	4	14,130,673	hetero	-31	tttctcttAATCCGTG---GGCTAAACagaaatat	tttctcttagaaatat	1
lig4-2-3	3	1,290,981	hetero	-5(-8+3)	aaatatcaATTTGATGaaaaataa	aaatatcaAAAaaaaataa	2
lig4-4-4	4	16,290,515	hetero	-14(-17+3)	acttcgacTTTGTTGTCTACTTCCATttgagctc	acttcgacGGGttgagctc	2
lig4-6-2	3	1,845,489	hetero	-8	tatatcaaTTTATGTTttcttgta	tatatcaattcttgta	2
lig4-1-6	5	25,337,855	hetero	-18	gtatagtcTAGTTGGA---AAGATGTAtattagtg	gtatagtctattagtg	2
lig4-9-3	3	11,276,246	hetero	-21	tctgaaaaGCAGTTTA---TCGTCTATgccctacg	tctgaaaagccctacg	2
lig4-2-3	2	5,650,588	hetero	-23	aaatggcgGATTCTTA---ACATGACCgaaatgag	aaatggcggaaatgag	2
lig4-9-3	2	5,048,684	hetero	-27	tctatataCTTAATAT---TTTCAATTctaaataa	tctatatactaaataa	2
lig4-2-3	3	15,756,609	hetero	-31	ctgcatgaCCTTATGC---TTCAAGATccagaagc	ctgcatgaccagaagc	2
lig4-4-4	2	5,610,873	hetero	-32	ataaagaaAATCAATA---TTAAAGAGaaatatcc	ataaagaaaaatatcc	2
lig4-2-3	1	6,026,400	hetero	-7(-9+2)	cactatgaGTAGGAGTTacaatagc	cactatgaCCacaatagc	3
lig4-1-6	2	4,037,518	homo	-3	tgatgtctTCCtccaacct	tgatgtcttccaacct	3
lig4-6-2	3	20,815,894	hetero	-3	gacgaaatGGAggaggagg	gacgaaatggaggagg	3
lig4-6-2	5	7,895,095	hetero	-6	ttttcaatAAGCAGaagaggag	ttttcaataagaggag	3
lig4-1-6	3	16,948,959	hetero	-27	attacgctGTTGACAC---GATGCACAgttttgga	attacgctgttttgga	3
lig4-1-6	3	16,825,322	homo	-27	atttccaaAACTGTGC---CACGTGTCaacagcgt	atttccaaaacagcgt	3
lig4-10-1	3	173,694	hetero	-30	ttggatgtGCAGCGAC---CGAGAGGAgcaaactg	ttggatgtgcaaactg	3
lig4-6-2	1	20,372,275	hetero	-31	attaaattACTGCATT---AGAGACTGactcttga	attaaattactcttga	3
lig4-4-4	3	21,514,441	homo	-39	gttttggtTTCATTGA---TGTCTGCCttcttccg	gttttggtttcttccg	3
lig4-2-3	5	25,065,565	homo	-258	attcaacaTTACACCC---TCCACAATttaaggtc	attcaacattaaggtc	3
lig4-5-2	2	4,001,280	hetero	-4	gaaaaagaTGTTtgtttaca	gaaaaagatgtttaca	4
lig4-4-4	1	13,676,768	hetero	-6	aactctgtTGGAGCtggatttc	aactctgttggatttc	4
lig4-4-4	2	14,741,827	homo	-6	gcagtttcTTCAAAttcacagt	gcagtttcttcacagt	4
lig4-4-4	2	7,024,279	hetero	-7	tttacataATAAAATataatata	tttacataataatata	4
lig4-4-4	2	5,915,547	hetero	-8	tttgatgcAACCAGGAaacccatt	tttgatgcaacccatt	4
lig4-1-6	1	16,659,690	homo	-11	gccatagtAAAAATGTTAAaaaagcat	gccatagtaaaagcat	4
lig4-3-5	5	6,441,521	homo	-13	gtgttagcAACCAAAGTTAAAaaccttt	gtgttagcaaccttt	4
lig4-6-2	4	1,516,777	hetero	-17	gcagtgttACCACAGGATGAGAGCGaccaaaag	gcagtgttaccaaaag	4
lig4-9-3	3	20,752,474	hetero	-20	aatcacacAAAATGAGAGAATATAATTAaaaaagga	aatcacacaaaaagga	4
lig4-1-6	5	17,975,116	hetero	-31	tacgtaccATCTATAT---TCTTACCAaaaaacaa	tacgtaccaaaaacaa	4
lig4-4-4	5	15,052,920	homo	-122	cgtggatgATAGGTAT---CTCATCTTgatggcta	cgtggatggatggcta	4
lig4-2-3	5	7,220,895	homo	-200	cttgaatcCTTGAGCA---GTGTGAAAccttaaca	cttgaatcccttaaca	4
lig4-7-4	2	2,346,228	hetero	-5	taaccttaGTAATgtaatact	taaccttagtaatact	5
lig4-1-6	1	20,107,265	homo	-6	ggccgcttTCTGCAtctgctca	ggccgctttctgctca	5
lig4-2-3	2	2,780,894	hetero	-19	gcccaataTTTTATATGCGGACGGATTttttaaac	gcccaatattttaaac	5
lig4-7-4	4	6,273,421	homo	-22	tctttttcTTGTATCA---AAATAATGttgtaaac	tctttttcttgtaaac	5
lig4-7-4	2	11,041,120	hetero	-31	taatatttTTTATAAA---AAAATCAAtttatttg	taatatttttatttg	5
lig4-5-2	2	16,210,037	homo	-36	aacggtttCTCAGGGA---CAGCTCTCctcagcga	aacggtttctcagcga	5
lig4-4-4	1	21,853,550	hetero	-6	cggttgtgAGAGAAagagattc	cggttgtgagagattc	6
lig4-7-4	3	16,938,484	hetero	-35	atataataAAGGAAAG---TGTCAACTaaggaatt	atataataaaggaatt	6
lig4-2-3	4	12,244,096	hetero	-70	atgtttgaTGCGGCGA---TGCACCTCgtttgaat	atgtttgagtttgaat	6
lig4-2-3	3	10,562,127	hetero	-7	atttgggcAGCTAAAagctaaaa	atttgggcagctaaaa	7
lig4-5-2	4	3,080,158	hetero	-12	gaaccatcAGCTAAGAGCAAagctaagtttc	gaaccatcagctaagtttc	7
lig4-2-3	2	9,504,635	homo	-35	gttttgatTTGATTTTTAGA---AGTGTACGttgattttagaa	gttttgatttgattttagaa	8
lig4-4-4	4	10,520,853	hetero	-27	ctattaagAAAAAAAAACGT---ATGAAACAaaaaaaaaaccaa	ctattaagaaaaaaaaaccaa	10
lig4-5-2	3	14,342,482	hetero	-4(-6+2)	cctcaataGACTGCtgtgcaag	cctcaataGTtgtgcaag	none
lig4-5-2	2	13,332,225	homo	-149	ttgggcctACTGGGCT---CGATCTCGctcctcca	ttgggcctctcctcca	none

Deletion >=2bp induced by 1,000 Gy of gamma rays in the wild type.

Sample	Chr#	Position	Zygosity	Deletion size	Original sequence	Altered sequence	Size of MH
1000Gy-1	2	12,179,281	hetero	-3	ttttTCCtaaa	tttttaaa	1
1000Gy-5	1	10,167,316	hetero	-3	agcgAAAacta	agcgacta	1
1000Gy-1	1	11,076,821	homo	-4	acctTCTCtggc	accttggc	1
1000Gy-3	3	10,826,722	hetero	-6	taaaATATTTacaa	taaaacaa	1
1000Gy-4	4	15,235,457	hetero	-11	ccatGCAC---TGAAgaaa	ccatgaaa	1
1000Gy-3	1	7,197,590	hetero	-2	tcgcATatat	tcgcatat	2
1000Gy-5	2	13,211,417	hetero	-2	ttttTAtatt	ttttatt	2
1000Gy-1	2	16,019,225	hetero	-2	aatgGTgtaa	aatggtaa	2
1000Gy-4	3	8,195,023	hetero	-2	ttacATatat	ttacatat	2
1000Gy-4	5	16,134,013	homo	-2	caatCAcata	caatcata	2
1000Gy-3	2	5,716,969	hetero	-3	aaaaAACaaat	aaaaaaat	2
1000Gy-1	1	28,135,505	hetero	-5	tgcaAGAACagtt	tgcaagtt	2
1000Gy-6	2	19,011,738	hetero	-6	ccagCGATGAggaa	ccagggaa	2
1000Gy-5	3	11,483,824	hetero	-12	ggctGATT---CAAAgagg	ggctgagg	2
1000Gy-2	3	6,870,451	hetero	-15	ttgcTGAG---TTTAtgga	ttgctgga	2
1000Gy-1	2	15,141,224	hetero	-21	actaTCGC---CCAGtcat	actatcat	2
1000Gy-4	4	4,134,219	homo	-28	ggctACTC---GAGAacgg	ggctacgg	2
1000Gy-5	1	12,496,337	hetero	-36	taatGGTA---TTCAggga	taatggga	2
1000Gy-6	5	24,032,804	hetero	-50	aaatTGAG---TGCGtggg	aaattggg	2
1000Gy-1	5	20,513,683	hetero	-53	ccttCTTA---TTTTattc	ccttattc	2
1000Gy-5	1	19,345,832	hetero	-75	aaatGATT---TCATatgc	aaatatgc	2
1000Gy-5	2	15,997,915	homo	-3	cttcCTTcttc	cttccttc	3
1000Gy-6	3	12,787,527	hetero	-3	tcagTAAtaag	tcagtaag	3
1000Gy-4	3	22,572,977	hetero	-4	tacaGATGgatt	tacagatt	3
1000Gy-3	1	9,647,544	hetero	-5	cgccGTTCTgttg	cgccgttg	3
1000Gy-2	3	14,661,020	hetero	-5	ggatAAAACaaag	ggataaag	3
1000Gy-6	1	26,676,916	hetero	-8	caccTTTCCAATtttt	cacctttt	3
1000Gy-1	4	2,195,126	homo	-12	tgaaGTGA---TGCAaggg	tgaaaggg	3
1000Gy-4	1	21,112,552	homo	-32	ttcgAGAA---AAGTagat	ttcgagat	3
1000Gy-4	1	30,038,887	homo	-39	aaagCCAT---TGCCccaa	aaagccaa	3
1000Gy-6	2	12,517,448	hetero	-12	acaaGAAGTTGGCTATgaagacga	acaagaag	4
1000Gy-3	3	1,638,950	hetero	-4,243	ttctTGAC---TAGTcttt	ttctcttt	4
1000Gy-4	3	4,806,512	hetero	-6	taaaTTTTCTttttctttt	taaatttt	6
1000Gy-3	3	11,025,457	hetero	-9	ctcaAAATATGTCaaatattt	ctcaaaat	6
1000Gy-5	5	25,466,761	homo	-22(-26+4)	atcaCAGC---ACTTagtg	atcaTGAAagtg	none
1000Gy-4	2	19,324,866	hetero	-2(-4+2)	gtgtTTTGgatc	gtgtCCgatc	none
1000Gy-6	1	8,842,910	homo	-14(-17+3)	taaaTGCA---TTTTaaat	taaaACAaaat	none
1000Gy-5	2	9,597,504	hetero	-2	tgcaTCcaat	tgcacaat	none
1000Gy-6	2	8,400,133	homo	-3	ttctCCAttga	ttctttga	none
1000Gy-2	5	15,982,783	hetero	-3	ttacCGAgaca	ttacgaca	none
1000Gy-5	5	11,287,515	homo	-4	tccaAATGcttg	tccacttg	none
1000Gy-4	4	13,855,106	hetero	-6	aagaGTGTGTaatt	aagaaatt	none
1000Gy-2	1	26,194,246	homo	-7	cctcCAATTGTatcc	cctcatcc	none
1000Gy-6	4	1,706,431	hetero	-7	tacgATGACTTtgcg	tacgtgcg	none
1000Gy-1	1	2,247,435	hetero	-88	cagaGCAT---TGAAtgaa	cagatgaa	none

Deletion >=2bp induced by 1,500 Gy of gamma rays in the wild type.

Sample	Chr#	Position	Zygosity	Deletion size	Original sequence	Altered sequence	Size of MH
1500Gy-7	1	9,505,179	homo	-7	taatATCAAAAactt	taatactt	1
1500Gy-2	1	22,507,370	hetero	-2	cagaACatgt	cagaatgt	1
1500Gy-7	2	2,842,720	hetero	-2	gactAGatat	gactatat	1
1500Gy-1	2	14,063,271	hetero	-7	ccggACATAGCaaaa	ccggaaaa	1
1500Gy-8	2	14,806,380	homo	-18	ctttTTGG---GGAGtcaa	cttttcaa	1
1500Gy-5	4	5,366,998	homo	-2	ggatAGatat	ggatatat	1
1500Gy-1	4	12,815,913	hetero	-2(-3+1)	caggTATtttt	caggAtttt	1
1500Gy-1	5	25,109,404	hetero	-2	aagaACaata	aagaaata	1
1500Gy-7	1	3,680,571	hetero	-7	ttttCACAAACaatc	ttttaatc	2
1500Gy-4	1	11,833,732	hetero	-2	ttgtGGggac	ttgtggac	2
1500Gy-6	1	22,631,942	hetero	-6	cgtgAAAACTaagg	cgtgaagg	2
1500Gy-7	2	253,730	homo	-2	tttgTTttta	tttgttta	2
1500Gy-6	2	17,301,892	hetero	-2	gtctCGcgcc	gtctcgcc	2
1500Gy-6	3	976,112	hetero	-73	ctgaTTAA---CAGAggag	ctgagagg	2
1500Gy-6	3	976,114	hetero	-73	ctgaTTAA---CAGAggag	ctgaggag	2
1500Gy-6	3	20,627,466	hetero	-6	taccTGGTTAtgat	tacctgat	2
1500Gy-7	4	2,498,000	hetero	-95	gaagCTCC---TGAGgaga	gaaggaga	2
1500Gy-5	4	2,773,412	homo	-4	atctATACatgc	atctatgc	2
1500Gy-3	4	2,910,168	hetero	-4	ggatTACAtaat	ggattaat	2
1500Gy-4	4	6,357,178	hetero	-5	aaaaAAAATaact	aaaaaact	2
1500Gy-7	4	6,650,274	hetero	-2	atatTAtatt	atattatt	2
1500Gy-5	4	14,720,528	hetero	-2	cgtgTTttgg	cgtgttgg	2
1500Gy-8	5	16,110,154	homo	-41(-42+1)	aatcTTTT---TATTttcg	aatcGttcg	2
1500Gy-6	5	24,140,044	hetero	-2	aacaATatat	aacaatat	2
1500Gy-7	2	6,498,349	hetero	-2	ttggACacaa	ttggacaa	2
1500Gy-4	1	11,667,262	hetero	-9	ggagCTTCCTTGAgttc	ggaggttc	3
1500Gy-5	1	19,529,880	hetero	-35	agcaTATA---CCACtatc	agcatatc	3
1500Gy-1	2	1,472,714	hetero	-5	ggagCATCAtatt	ggagtatt	3
1500Gy-3	2	2,359,919	homo	-3	tcctCCAccac	tcctccac	3
1500Gy-3	2	9,043,691	homo	-27(-40+13)	ttgaTTCA---TACTttct	ttgaCCACCTATATATAttct	3
1500Gy-2	2	13,873,663	hetero	-3	tcttCAAcaac	tcttcaac	3
1500Gy-3	2	16,343,308	homo	-8	tcgcGTAATTTGgtat	tcgcgtat	3
1500Gy-1	2	18,736,554	hetero	-4	agatATTGatta	agatatta	3
1500Gy-2	2	19,279,820	hetero	-7	aatgCTGCTCActga	aatgctga	3
1500Gy-6	3	6,911,454	hetero	-7	tttcCTTTGCTcttc	tttccttc	3
1500Gy-8	3	15,616,715	hetero	-3	tagaAAGaagg	tagaaagg	3
1500Gy-7	4	5,445,597	hetero	-4	gccaTCTCttct	gccattct	3
1500Gy-6	5	1,747,968	hetero	-17	ggtcCCAC---GCTTccag	ggtcccag	3
1500Gy-7	5	4,223,065	hetero	-15	tggaGCTG---TAATgctt	tggagctt	3
1500Gy-3	5	11,995,223	hetero	-18	tgtcCCTA---CTTGcctg	tgtccctg	3
1500Gy-7	5	24,242,485	hetero	-4	aaggATCAatct	aaggatct	3
1500Gy-4	5	24,645,530	hetero	-6	actaACACTTacat	actaacat	3
1500Gy-4	2	7,940,876	hetero	-22	acctATGTCA---CAGAatgtgg	acctatgt	4
1500Gy-7	3	7,801,573	hetero	-6	taaaTAAATGtaaa	taaataaa	4
1500Gy-6	3	19,002,792	hetero	-23	tggcATCTTA---CATAatctca	tggcatct	4
1500Gy-4	5	24,366,197	hetero	-10	ttggATTTAAAAGTatttgt	ttggattt	4
1500Gy-1	3	10,503,045	hetero	-10	agtgATATATATATatatatatatat	agtgatat	10
1500Gy-4	1	2,701,315	homo	-14	ctttCTCC---CTTGaatc	ctttaatc	none
1500Gy-3	1	9,776,373	hetero	-31(-37+6)	caaaCCAG…GTTCtgaa	caaaAGCATAtgaa	none
1500Gy-1	1	12,333,406	hetero	-22(-25+3)	ccacGAGA---TTCAaaaa	ccacTTTaaaa	none
1500Gy-8	1	21,951,980	hetero	-2	gcttTAacag	gcttacag	none
1500Gy-2	1	22,194,972	hetero	-14	cactCCTG---TACAaaca	cactaaca	none
1500Gy-3	2	7,084,629	homo	-17	ttttTTTT---GTTGaaaa	ttttaaaa	none
1500Gy-4	2	8,998,398	hetero	-2(-3+1)	atttGACatac	atttAatac	none
1500Gy-8	2	11,731,456	homo	-21	tataATGT---TTGTttat	tatattat	none
1500Gy-2	2	13,754,919	hetero	-3	ttccTCGatgc	ttccatgc	none
1500Gy-4	2	18,008,523	hetero	-7	gaccTGCTTGAaaac	gaccaaac	none
1500Gy-4	3	3,521,042	homo	-22(-44+2)	tgccACCA---CGACgaca	tgccATgaca	none
1500Gy-7	3	3,923,551	hetero	-24	tttgGAGA---AGTAcacg	tttgcacg	none
1500Gy-7	3	12,678,300	homo	-2	cacaCTtcct	cacatcct	none
1500Gy-3	3	17,738,880	hetero	-14	ggctCAGC---AAGGatgc	ggctatgc	none
1500Gy-1	3	18,575,840	homo	-4	ttcaTGCGcttt	ttcacttt	none
1500Gy-8	3	20,876,786	hetero	-7	cccaCAAGCCCtatc	cccatatc	none
1500Gy-7	4	6,828,713	hetero	-4	tcgaCCTCtaaa	tcgataaa	none
1500Gy-3	4	9,693,523	hetero	-8	aaatCTGTGCACgtag	aaatgtag	none
1500Gy-5	4	12,583,485	hetero	-5	gaaaATTGCtctt	gaaatctt	none
1500Gy-1	4	14,544,469	hetero	-2	ggctCAtcga	ggcttcga	none
1500Gy-5	4	15,144,092	hetero	-2	ctgcCTgcat	ctgcgcat	none
1500Gy-8	5	1,458,449	homo	-2	cacgAAggca	cacgggca	none
1500Gy-7	5	7,188,136	hetero	-6(-10+4)	aggaGGACTTGCTGttga	aggaGATAttga	none
1500Gy-1	5	22,602,276	hetero	-8	tggcCAAGTCTTtctt	tggctctt	none

Deletion >=2bp induced by 125 Gy of carbon ions in the wild type.

Sample	Chr#	Position	Zygosity	Deletion size	Original sequence	Altered sequence	Size of MH
125-12-1	2	7,156,412	homo	-2	taatAAagtt	taatagtt	1
125-5-1	1	26,920,198	hetero	-5	ttaaGAGATggta	ttaaggta	1
125-5-1	5	25,068,035	hetero	-5	ttcaAATCGactc	ttcaactc	1
125-5-1	2	6,937,742	hetero	-6	gtcaTGCTTCtcta	gtcatcta	1
125-5-1	3	9,239,983	homo	-8	ttacTCCAAGTGtaga	ttactaga	1
125-12-1	5	3,487,785	hetero	-14	gaatATAA---CAGGaaga	gaataaga	1
125-6-1	5	24,360,523	hetero	-23	tagtAAAG---ACTAaccg	tagtaccg	1
125-12-1	2	364,655	hetero	-26	tgaaACCA---AAATatca	tgaaatca	1
125-2-2	2	14,419,918	hetero	-2	tgagTTtttt	tgagtttt	2
125-10-5	3	16,755,265	hetero	-2	gaaaTCtcaa	gaaatcaa	2
125-5-1	3	5,294,313	homo	-2	tcaaACacat	tcaaacat	2
125-2-2	4	17,312,251	hetero	-3	atttTAAtaga	attttaga	2
125-5-1	3	4,153,732	homo	-3	cttgCTActca	cttgctca	2
125-6-1	4	6,696,328	homo	-3	aatgTAAtagc	aatgtagc	2
125-2-2	5	18,767,398	hetero	-6	gtgaTGGTTGtgac	gtgatgac	2
125-4-1	5	15,405,223	hetero	-6	tttgCTAAACcttg	tttgcttg	2
125-6-1	3	15,878,581	hetero	-6	gaaaAGGGAGagat	gaaaagat	2
125-12-1	2	7,709,941	homo	-6	tactATGAACatta	tactatta	2
125-6-1	1	4,623,134	homo	-12	tgttTCTA---AACAtcaa	tgtttcaa	2
125-2-2	5	20,891,510	hetero	-17	agtgATTT---GTACatct	agtgatct	2
125-10-5	2	6,248,477	hetero	-22	ggagTTTT---CTCTttgt	ggagttgt	2
125-2-2	4	8,128,297	homo	-26	ctcaGTTG---GAAGgtaa	ctcagtaa	2
125-5-1	4	4,902,653	homo	-1,264	attcACTA---TAGAacgg	tcacacgg	2
125-5-1	1	3,133,880	hetero	-2(-3+1)	agtaGTCgtcg	agtaTgtcg	3
125-10-5	3	6,821,869	hetero	-11(-12+1)	tcttGAAG---ATCAagca	tcttTagca	3
125-10-5	4	15,014,172	hetero	-3	aattAAAaaag	aattaaag	3
125-12-1	4	10,472,631	hetero	-3	gatgCTCctcc	gatgctcc	3
125-5-1	5	1,091,474	hetero	-3	actgCTTcttg	actgcttg	3
125-2-2	1	9,124,997	hetero	-5	cgatAATCAgaatatca	cgatgaat	3
125-5-1	1	28,799,771	hetero	-7	gaaaTGATACCtgag	gaaatgag	3
125-12-1	2	2,546,303	hetero	-9	aatgATGGCGACAatga	aatgatga	3
125-6-1	2	13,026,798	homo	-9	tagtTTTGTTTGGtttt	tagttttt	3
125-5-1	3	5,002,764	homo	-32	tcctTTGG---TGATtgat	tccttgat	3
125-5-1	5	1,560,738	hetero	-4	ttatAAGAaagaaa	ttataaga	4
125-2-2	4	14,147,272	hetero	-5	atctTCATCttcaactt	atctttca	4
125-2-2	1	24,726,936	hetero	-8	ttatTACAGATCtacaatac	ttattaca	4
125-6-1	5	13,796,775	homo	-8	gctaAACTAAAAccca	gctaccca	4
125-5-1	2	12,559,488	hetero	-17	gagtTCATCC---CTACtcataa	gagttcat	4
125-4-1	3	11,165,929	homo	-8	agctACAAAATCacaaaggg	agctacaa	5
125-5-1	1	11,886,904	homo	-13	ctagAGAAgTCT---TGCTagaagctc	ctagagaa	5
125-4-1	5	24,449,837	hetero	-32	atgcGAATATCA---AAGTgaataacc	atgcgaat	5
125-4-1	1	10,335,193	hetero	-23	gcaaAGAGAGAT---ACAGagagagag	gcaaagag	7
125-10-5	3	11,170,188	hetero	-5(-6+1)	cctaAACCCGtcat	cctaTtcat	none
125-5-1	3	14,639,518	homo	-4(-5+1)	ttacCAGCTgtct	ttacAgtct	none
125-10-5	1	25,115,764	hetero	-2	ttcaCTaact	ttcaaact	none
125-12-1	3	21,581,283	homo	-2	gtggATtaat	gtggtaat	none
125-2-2	4	10,723,555	homo	-2	accaGTatgc	accaatgc	none
125-6-1	3	17,786,949	hetero	-2	aggaCCaaag	aggaaaag	none
125-5-1	2	13,824,297	hetero	-3	ggttATGtttt	ggtttttt	none
125-5-1	5	16,579,478	homo	-3	gaccAATgtac	gaccgtac	none
125-6-1	3	8,041,753	hetero	-3	tattTTGaaaa	tattaaaa	none
125-4-1	5	16,424,864	hetero	-5	tttcCGGCTaatt	tttcaatt	none
125-5-1	1	6,253,689	hetero	-5	atgaATTAGgtgc	atgagtgc	none
125-5-1	1	26,072,351	hetero	-6	taagAGAGGAcaag	taagcaag	none
125-6-1	5	25,905,384	hetero	-7	tgtaTTTGAATcaat	tgtacaat	none
125-5-1	4	18,242,556	hetero	-8	gcagAATCAGGTtctg	gcagtctg	none
125-12-1	1	824,389	homo	-9	agacGTCTAATTTtttt	agactttt	none
125-10-5	5	15,422,503	hetero	-12	gaaaAAAA---GAATcacg	gaaacacg	none
125-12-1	5	17,528,084	homo	-17	gccgGAGG---GACCtagt	gccgtagt	none
125-2-2	2	14,816,650	homo	-26	cagtCAGC---TGACttct	cagtttct	none
125-2-2	4	9,618,651	homo	-36	ataaGAAG---GCTCttac	ataattac	none
125-5-1	2	14,944,754	hetero	-37	tcccTTTT---TAGCaaac	ctcccaaa	none
125-2-2	5	296,639	hetero	-44	actcCGTT---CAGAgtcg	actcgtcg	none
125-4-1	5	4,935,416	homo	-52	aactTAAG---TACAaact	aactaact	none
125-10-5	4	11,649,411	hetero	-144	ctttCTTT---GTAAttgt	ctttttgt	none
125-10-5	1	29,787,264	hetero	-308	atatAGAA---CTACgagc	atatgagc	none
125-12-1	2	19,344,169	hetero	-6,313	agatACAG---ACATtttt	agattttt	none
125-6-1	2	6,349,896	homo	-245,416	tataTTGG---TCCGagag	tataagag	none

Deletion >=2bp induced by 175 Gy of carbon ions in the wild type.

Sample	Chr#	Position	Zygosity	Deletion size	Original sequence	Altered sequence	Size of MH
175-6-1	4	5,470,763	homo	-2	tccgAAacaa	tccgacaa	1
175-2-4	1	2,878,334	hetero	-4	tataGTTTgatt	tatagatt	1
175-12-1	5	25,748,360	homo	-5	tgacTAATTtttt	tgactttt	1
175-6-1	1	25,253,238	hetero	-6	caccACCGTTaaga	caccaaga	1
175-12-1	3	15,239,723	hetero	-7	cctaTAAATACtgaa	cctatgaa	1
175-5-1	2	10,888,982	homo	-8	atgtATTGTTAGaaat	atgtaaat	1
175-5-1	5	26,591,541	hetero	-8	tttgGCTCACGAgatg	tttggatg	1
175-6-1	4	12,953,725	hetero	-13	tggcTCAA---ACGTtagc	tggctagc	1
175-2-4	4	12,174,699	homo	-16	acagAGAT…TAAAactc	acagactc	1
175-6-1	1	6,417,811	hetero	-18	caacACTT---CAAGaatt	caacaatt	1
175-6-1	2	3,849,780	homo	-28	cttgTTAA---CTAAtgac	cttgtgac	1
175-2-4	5	24,286,276	hetero	-2	caggTAtata	caggtata	2
175-5-1	2	395,716	homo	-2	atctAGagat	atctagat	2
175-5-1	3	7,522,459	hetero	-2	ttcaATatct	ttcaatct	2
175-4-1	1	22,115,903	hetero	-3	cctaACTacat	cctaacat	2
175-4-1	4	15,364,201	hetero	-4	gaggTAACtaca	gaggtaca	2
175-6-1	1	24,901,914	hetero	-12	ttaaATGA---GATGataa	ttaaataa	2
175-4-1	3	8,187,494	hetero	-18	atatAGTT---AAAAgctt	atatgctt	2
175-4-1	5	20,924,511	homo	-29	attgGTCC---TTAAgttc	attggttc	2
175-1-4	5	26,505,636	homo	-36	cggtAGAA---CGGCagga	cggtagga	2
175-5-1	4	10,476,322	hetero	-7(-10+3)	aaaaGTTGTTTTGTttaa	aaaaAACttaa	3
175-12-1	5	6,481,553	hetero	-3	gcaaATTaatg	gcaaaatg	3
175-2-4	4	4,274,350	homo	-3	tctaCATcatc	tctacatc	3
175-4-1	4	616,130	homo	-3	atatGAAgaaa	atatgaaa	3
175-5-1	4	4,180,450	hetero	-3	atatGCAatga	atatatga	3
175-6-1	1	5,341,405	hetero	-3	tcaaTGTtgtt	tcaatgtt	3
175-1-4	1	20,186,405	hetero	-4	accaAAACcact	accacact	3
175-5-1	1	17,303,879	hetero	-4	agagGTACgtaa	agaggtaa	3
175-6-1	4	18,553,820	hetero	-4	gagtTTCCttca	gagtttca	3
175-2-4	3	12,794,444	hetero	-5	aatcTTTTTtttta	aatcttta	3
175-5-1	1	5,668,629	homo	-13	tctgATCG---GGAAatca	tctgatca	3
175-6-1	4	11,821,711	hetero	-24	tcgaATGT---TCGCatgc	tcgaatgc	3
175-2-4	3	13,531,918	hetero	-32	gaacCGTA---CTGTcgtt	gaaccgtt	3
175-5-1	3	17,667,864	homo	-166	ttcaTCTC---TGATctta	ttcactta	3
175-5-1	1	5,166,926	homo	-2	agttCCttcc	agttttcc	4
175-4-1	3	9,031,896	hetero	-4	ttagTTATttatactg	ttagttat	4
175-5-1	1	27,457,660	homo	-4	tgtcCTAActaa	tgtcctaa	4
175-4-1	4	13,728,384	homo	-5	atccACAAAacaaccta	atccacaa	4
175-2-4	1	11,770,737	hetero	-13	tactCCAA---GTTCcata	tactcata	4
175-5-1	3	1,457,700	hetero	-16	cttgTCAAGACA---ATGAtcaatgta	cttgtcaa	4
175-1-4	1	8,909,976	hetero	-30	catgTCGGTT---AATTtcggga	actgtcgg	4
175-2-4	3	18,497,940	hetero	-12	gatgTGAAGCCG---GTAAtgaagaga	gatgtgaa	5
175-5-1	4	11,621,149	hetero	-16	aaatTTACTTCA---AAAAttactaat	aaatttac	5
175-4-1	3	18,753,702	hetero	-25	atccAGTTTTTA---CAAAagtttgaa	atccagtt	5
175-6-1	1	18,637,436	homo	-12	aatgTTGTCTCT---ACCCttgtctcc	aatgttgt	7
175-2-4	1	8,437,322	hetero	-44(-45+4)	caatCCTA---TGCAgata	caatTGATgata	none
175-4-1	4	10,795,489	homo	-4(-5+1)	gaacACATGtgtt	gaacGctgtt	none
175-5-1	1	12,593,613	homo	-2(-3+1)	aacaAATccca	aacaCccca	none
175-5-1	4	6,888,504	hetero	-10(-11+1)	attcTTTA---TTTCgtta	attctAgtta	none
175-1-4	5	16,344,361	homo	-2	acagAAcaca	acagcaca	none
175-5-1	5	13,307,876	homo	-2	ctgtTCacgc	ctgtacgc	none
175-6-1	1	4,530,659	hetero	-2	ctcaGCtaac	ctcataac	none
175-6-1	1	10,940,869	hetero	-2	gagcGTcatt	gagccatt	none
175-12-1	3	11,406,835	hetero	-3	agaaTTGcctt	agaacctt	none
175-1-4	2	2,788,013	homo	-3	actaAACctac	actactac	none
175-1-4	5	16,617,906	homo	-3	ctggTAAgcat	ctgggcat	none
175-12-1	5	18,900,692	hetero	-4	agacCAAAaaaa	agacaaaa	none
175-12-1	5	11,147,410	hetero	-5	ttccTAACAagca	ttccagca	none
175-5-1	2	16,163,504	homo	-6	gaagGCAAACtagg	gaagtagg	none
175-6-1	5	9,293,322	homo	-6	atgcAGGCGAttta	atgcttta	none
175-1-4	4	4,704,733	homo	-7	tcccTAAGCGAgttc	tcccgttc	none
175-2-4	2	12,367,411	hetero	-8	tggaACTCTTTTtttt	tggatttt	none
175-2-4	5	18,926,429	homo	-8	attaGATAACATatag	attaatag	none
175-4-1	1	5,652,997	hetero	-8	tgctTTGTTTGGacaa	tgctacaa	none
175-5-1	1	12,144,842	homo	-13	ttcaAGCT---GTTCtgtt	ttcatgtt	none
175-5-1	2	16,319,558	homo	-14	gggtGTAA---ATAAaata	gggtaata	none
175-12-1	3	4,087,237	hetero	-37	tgtaAGCT---TGTTgaag	tgtagaag	none
175-2-4	5	2,024,079	homo	-45	gcatTCGA---CCAGgatt	gcatgatt	none
175-2-4	1	17,258,954	hetero	-67	ggcaGCGT---AGCAttac	ggcattac	none
175-4-1	5	15,108,168	homo	-281,933	atctTCTC---AAAAacta	atctacta	none
